# Immunopeptidomics of *Salmonella enterica* Serovar *Typhimurium*-Infected Pig Macrophages Genotyped for Class II Molecules

**DOI:** 10.3390/biology13100832

**Published:** 2024-10-16

**Authors:** Carmen Celis-Giraldo, Carlos F. Suárez, William Agudelo, Nieves Ibarrola, Rosa Degano, Jaime Díaz, Raúl Manzano-Román, Manuel A. Patarroyo

**Affiliations:** 1 Veterinary Medicine Programme, Universidad de Ciencias Aplicadas y Ambientales (U.D.C.A), Bogotá 111166, Colombia; ccelis@udca.edu.co (C.C.-G.); jaimdiaz@udca.edu.co (J.D.); 2 PhD Programme in Tropical Health and Development, Doctoral School “Studii Salamantini”, Universidad de Salamanca, 37007 Salamanca, Spain; 3 Grupo de Investigación Básica en Biología Molecular e Inmunología (GIBBMI), Fundación Instituto de Inmunología de Colombia (FIDIC), Bogotá 111321, Colombia; cfsuarezm@gmail.com (C.F.S.); wagudelos@gmail.com (W.A.); 4 Centro de Investigación del Cáncer e Instituto de Biología Molecular y Celular del Cáncer (IBMCC), CSIC-Universidad de Salamanca, 37007 Salamanca, Spain; nibarrola@usal.es (N.I.); romade@usal.es (R.D.); 5 Infectious and Tropical Diseases Group (e-INTRO), IBSAL-CIETUS (Instituto de Investigación Biomédica de Salamanca—Centro de Investigación de Enfermedades Tropicales de la Universidad de Salamanca), Pharmacy Faculty, Universidad de Salamanca, 37007 Salamanca, Spain; rmanzano@usal.es; 6 Microbiology Department, Faculty of Medicine, Universidad Nacional de Colombia, Bogotá 111321, Colombia

**Keywords:** *Salmonella typhimurium*, immunopeptidomics, peptide, vaccine, pig

## Abstract

Salmonellosis is a zoonotic infection that has a major impact on human health; consuming contaminated pork products is the main source of such infection. Vaccination responses to classic vaccines have been unsatisfactory; that is why peptide subunit-based vaccines represent an excellent alternative. Immunopeptidomics was used in this study as a novel approach for identifying antigens coupled to major histocompatibility complex class II molecules. Three homozygous individuals having three different haplotypes (Lr-0.23, Lr-0.12, and Lr-0.21) were thus selected as donors; peripheral blood macrophages were then obtained and stimulated with *Salmonella typhimurium* (MOI 1:40). Although similarities were observed regarding peptide length distribution, elution patterns varied between individuals; in total, 1990 unique peptides were identified as follows: 372 for Pig 1 (Lr-0.23), 438 for Pig 2 (Lr.0.12) and 1180 for Pig 3 (Lr.0.21). Thirty-one *S. typhimurium* unique peptides were identified; most of the identified peptides belonged to outer membrane protein A and chaperonin GroEL. Notably, 87% of the identified bacterial peptides were predicted in silico to be elution ligands. These results encourage further in vivo studies to assess the immunogenicity of the identified peptides, as well as their usefulness as possible protective vaccine candidates.

## Simple Summary

Subunit vaccines based on peptides as minimal immunogenic epitopes/antigens can induce highly specific immune responses without triggering adverse reactions. Here, *Salmonella enterica serovar Typhimurium* peptides were identified in the immunopeptidome of porcine macrophages genotyped for different class II molecules that were stimulated with the bacteria. Immunopeptidomics and mass spectrometry approaches were used for peptide identification, along with two predictors for immunoinformatics analysis. Differences between individuals were observed concerning length, elution preferences, and elution predictions. Thirty-one bacterial peptides were identified; outer membrane protein-A and chaperonin GroEL protein had the highest number of peptides. Since proteins having the highest number of peptides have been suggested as candidate antigens for vaccines, all the bacterial peptides identified can be considered promising candidate epitopes upon determining their immunogenicity.

## 1. Introduction

Salmonellosis is a zoonotic bacterial disease that is distributed worldwide; it affects around 1.3 billion people every year [1]. *Salmonella enterica* subsp. enterica serovar Typhimurium (*S. typhimurium*) is the serotype having cosmopolitan distribution [2]. The main source of transmission in humans is associated with the consumption of contaminated animal products such as eggs, poultry [3], and pork, the latter being particularly important [4].

*Salmonella typhimurium* infection in pigs is mostly asymptomatic. Individuals excrete the microorganism into the environment where it remains for long periods of time due to its presence in lymphoid organs and the intestines; carcasses thus frequently become contaminated in processing plants [5]. Symptomatic animals have yellow diarrhoea (rarely with blood), are dehydrated, consume less food, have a fever, and starve; predisposing factors such as poor hygiene in herds and viral diseases promote immunosuppression and disease development [6].

The main treatment strategy consists of using antibiotics; however, the emergence of resistance mechanisms has been increasingly observed, as well as an increase in multi-resistant strains (MDRs) such as *S. typhimurium ST3131* [7]. Vaccination is a recognised strategy for controlling the disease in humans and animals [8]. Both humoral and cellular immune responses must be stimulated to ensure protection, as this is a facultative intracellular microorganism [9]. The oral live attenuated Ty21a and the parenteral Vi polysaccharide vaccines are currently available for humans [10]. Attenuated or inactivated vaccines have been reported for use with pigs and are available in some countries, some containing more than one serovar [11]. De la Cruz et al. (2017) reported similar efficacy regarding attenuated and inactivated vaccines; response to colonisation and the elimination of the microorganism was variable, with greater protection being observed when both types of vaccines were combined [12]. Other Salmonellosis vaccines are based on subunits—proteins or peptides—with several advantages over the whole vaccine [9].

As immunopeptidomics is a state-of-the-art bioanalytical method it enables identifying a particular set of peptides presented by major histocompatibility complex (MHC) molecules [13]. This technique has been considered a necessary tool for identifying epitopes for developing vaccines against cancer in recent years (along with bacterial, viral, and parasitic infections) [13,14]. Mass spectrometry (MS) is an analytical technique having great sensitivity and specificity [15]; thousands of unique peptides can be identified in a single, unbiased analysis [16]. MHC molecules can be isolated from cell or tissue lysis using allele-specific [17] or pan-specific antibodies [18] or using an affinity tag system [19]. Regarding MHC-II analysis, identification in the vast majority of cases comes from studies involving human cells [20]; very few studies have dealt with other species, such as rodents [21] and bovines [22]. At the time of preparing this paper, only one immunopeptidomics study was found concerning class I molecules in porcine species [23].

Regarding MHC-II, peptides from the microorganism can be identified on infected antigen-presenting cell surfaces (e.g., macrophages, dendritic cells), thereby enabling qualitative and quantitative visualisation of the immunopeptidome [14]. Hundreds or thousands of peptides presented by these molecules can be identified in the data obtained from the immunopeptidome, thereby facilitating the analysis of antigen presentation pathway properties [24]. Such data are useful for improving neural network performance for predicting MHC ligands and naturally presented epitopes [25].

MHC molecules in pigs are called swine leucocyte antigen (SLA) [26,27,28]; the set of genes making up SLA is characterised by being highly variable [29]. SLA-DRB1 and SLA-DQB1 are the most polymorphic class II genes; they are associated with resistance or susceptibility to diseases, as well as vaccine responses [30]. It is worth noting that MHC’s trans-species nature implies that certain of these molecules’ sequence motifs are conserved in different species. This phenomenon arises from the MHC’s ability to evolve in response to similar pathogens, resulting in peptide presentation, which is not limited to a single species. Consequently, MHC alleles from distinct species may have a similar repertoire of epitopes, grouping them into supertypes. Molecular convergence also influences this process as different species develop MHC molecules which, despite evolutionary divergence, have similar functions regarding antigen presentation. Such convergence enables different species to share immune response aptitude against common pathogens [20,31,32]. These findings have significant implications for zoonotic disease research and for understanding cross-species immune responses.

This research has resulted from a thorough study on the use of immunopeptidomics for identifying presented peptides in *Salmonella enterica* serovar *Typhimurium*-infected porcine macrophages. Unlike traditional methods, which often rely on in vitro peptide binding assays or computational predictions alone, immunopeptidomics directly analyses peptides naturally presented by antigen-presenting cells. This ensures more precise identification of epitopes relevant to triggered immune responses. This research focused on genotyping class II molecules in pig macrophages to find potential vaccine candidates for enhancing immune responses. This work’s significant contribution lies in identifying 31 unique peptides, primarily from outer membrane protein A and chaperonin GroEL; the NetMHCIIpan-4.0 neural network was used for predicting/demonstrating strong binding affinity. Genotyped alleles were introduced into computational models to improve MHC II binding affinity prediction, thus offering a robust framework for future in vivo validation. Our approach has the potential for designing targeted peptide-based vaccines for various pathogens, enhancing immune response specificity and efficacy for different species. Moreover, the findings underscore immunopeptidomics’ usefulness for rational vaccine design by targeting specific epitopes. This approach could lead to developing more effective peptide-based, viral vectors or RNA vaccines against zoonotic diseases like salmonellosis, thereby improving both animal and human health outcomes [33,34].

## 2. Materials and Methods

### 2.1. Experimental Animals

Three adult breeding pigs were used for this research, two 2–4-year-old females from the SuperMom 52 line and a 7-year-old male from the commercial PIC line; the animals were kept at the Universidad de Ciencias Aplicadas y Ambientales’ (U.D.C.A) El Remanso academic unit in Bogotá, Colombia, during the study.

### 2.2. Genotyping SLA-DRB1 and SLA-DQB1

Three homozygous individuals having three different alleles were selected, considering previously reported results of an SLA-DRB1 gene variability study in pigs from the El Remanso academic unit (U.D.C.A) [35]. The SLA-DRB1 gene’s exon 2 was amplified using previously reported primers: forward 5′-GTC CAC GCA GCG CAT TTC TT-3′ [36] and reverse 3′-ACA CAC ACT CTG CCC CCC G-5′ [37]; the amplified fragment was 328 bp. The reaction volume was 25 μL, distributed as follows: 7 μL nuclease-free water, 12.5 μL Kodaq 2x PCR Master Mix, 1.25 μL for each primer [10 μM], and 3 μL genomic DNA. The amplification conditions consisted of an initial denaturing at 94 °C for 3 min, 35 cycles consisting of denaturing at 94 °C for 30 s, annealing at 68.2 °C for 30 s, and extension at 72 °C for 1 min, followed by a final extension step at 72 °C for 5 min. All PCR products were amplified in 2 independent reactions, revealed on 2% agarose gel, and then purified and sent to Sanger sequencing.

The SLA-DQB1 gene was genotyped; this involved verifying that the stored genomic material of the 3 previously selected individuals met the quality parameters to be used in polymerase chain reaction (PCR) assays. The PCR assays also involved amplifying exon 2, using a pair of primers reported in the pertinent literature: [38] forward *(DQB1F-119)* 5’-GCGGCGGGTTTCAGGTGGATG-3´ and reverse *(DQB1R+295)* 5´-aaccctcactaaagACCCACTCTCTCYGCGCGGWGTCTC-3´; amplified fragment length was 478 bp. Modifications made to the previously reported methodology can be described briefly as follows [38]: The high-fidelity Kodaq 2X PCR MasterMix enzyme was used; the reaction volume was 25 µL, distributed as 7 µL nuclease-free water, 12.5 µL Master Mix enzyme, 1.25 µL for each primer [10 µM] and 3 µL genomic DNA (concentration varied from 50 to 100 μg/μL). Amplification conditions were similar to those described for the other PCR; the annealing temperature was modified: 66.4 °C for 30 s.

The amplicons were confirmed on 2% agarose gel and purified by enzymatic reaction using ExoSAP-IT Express PCR Cleanup Reagent, following the manufacturer’s recommendations. Briefly, an 8.8 µL volume was added per PCR tube; a 15 min 37 °C cycle was used for degrading primer and nucleotide remnants and a 15 min 80 °C cycle for inactivating the enzyme. The products were then identified on 2% agarose gel. The Sanger method was used for sequencing the products in both directions. The CLC Genomics Workbench (QIAGEN) was used for allele alignment, using exon 2 SLA-DQB1*01:01allele as reference. The BLAST server was used for analysing allele identity (https://blast.ncbi.nlm.nih.gov/; accessed on 12 March 2022).

### 2.3. Macrophage Differentiation and In Vitro Infection

Jugular venipuncture was used to obtain peripheral blood monocytes, which were then converted to macrophages, using a previously reported process [39]. Briefly, the blood was spun at 1200× *g* for 20 min, and cells were separated by density gradient using a Ficoll–Paque medium (Histopaque), and 10^6^ monocytes/well were sown on 24-well plates for 96 h at 37 °C with 5% CO_2_, until maturation_._ The *Salmonella enterica* serovar typhimurium (ATCC 14028) used in this study was generously donated by the Instituto Colombiano Agropecuario (ICA). A 1:40 multiplicity of infection (MOI) had been determined in previous assays as the optimum MOI for stimulating macrophages; the same conditions for obtaining, inactivating, and labelling bacteria were thus used, as previously reported [39].

A total of 10^9^ bacteria-stimulated macrophages were obtained from each pig at 1:40 MOI for 20 h; the macrophages were then removed from culture plates using trypsin (7 min). SLA-DRB1 expression was analysed by flow cytometry, following the previously reported protocol.

### 2.4. Isolating SLA-Class II-Peptide Immunocomplexes

Previously described conditions were used for isolating peptides presented by each donor pig’s class II molecules [39]. Briefly, 10^9^ cells/mL were lysed with buffer containing 20 mM Tris-HCl, pH 8, 1% CHAPS, and 5 mM EDTA. Protease inhibitors were added, and the mixture was incubated at 4 ◦C in a tumble shaker for 1 h and then spun at 21,000× *g* for 20 min. The supernatant was incubated in an affinity column at 4 °C in a tumble shaker overnight. A 3 mg mAb-L243/total immunoaffinity column coupled to protein A was used for capturing the immunocomplexes. Following incubation at 4 °C overnight, the column was washed with 10 volumes of buffers 1 and 2, as described previously [39], with buffer 1 (0.005% (*v*/*v*) consisting of IGEPAL CA-630; 50 mM Tris, pH 8.0; 150 mM NaCl; 5 mM EDTA; 100 μM PMSF; and 1 μg/mL pepstatin A. Buffer 2 consisted of 50 mM Tris, pH 8.0, and 150 mM NaCl; buffer 3 comprised 50 mM Tris, pH 8.0, and 450 mM NaCl; and buffer 4 comprised 50 mM Tris, pH 8.0 [27]. MHC II molecules were eluted with 5 volumes of 10% acetic acid, obtaining 5 fractions per sample in Thermo Scientific Pierce Low Protein Binding Microcentrifuge Tubes. All fractions were lyophilised and sent at −20 °C to the Universidad de Salamanca’s Cancer Research Centre’s (CIC) Proteomics Service for liquid chromatography–tandem mass spectrometry (LC-MS/MS) analysis.

### 2.5. Mass Spectrometry Analysis

A nano-UHPLC system (NanoElute, Bruker Daltonics, Bremen, Germany) coupled to a hybrid trapped ion mobility–quadrupole time-of-flight mass spectrometer TimsTOF-Pro (Bruker Daltonics, Germany) via a modified nano-electrospray ion source (Captive Spray, Bruker Daltonics, Germany) was used for reversed-phase LC-MS/MS analysis. Peptides were dissolved in 0.1%TFA/1%ACN, loaded onto a Neo Trap Cartridge (PepMap, Thermo Scientific, Waltham, MA, USA) and separated on a Aurora C18 1.9 um 75ID 25 cm column (IonOpticks, Fitzroy, Victoria, Australia) at 50 °C, using 90 min gradients from 1% to 17% ACN/0.1 FA in 60 min, 17% to 25% in 30 min and 25% to 37% in 10 min, at a 400 nL/min flow rate. MS acquisition was run in data-dependent acquisition (DDA) mode with parallel accumulation serial fragmentation (PASEF) for improving proteomics analysis speed, sensitivity, and comprehensiveness. One full MS and 10 PASEF MS/MS frames were acquired for each topN acquisition cycle, resulting in 1.9 s cycle time. The parameters were 100–1700 *m*/*z* range, 1/K0 mobility range of 0.6–1.6 Vs/cm^2^, and 166 ms accumulation time, resulting in 1.9 s cycle time. A polygon filter was used in the low *m*/*z* and ion mobility area to exclude singly charged ions below 700 *m*/*z* from PASEF precursor selection. The parameters used during MS/MS were 1000 intensity threshold, 20,000 target intensity, and 0 to 5 charge range.

### 2.6. LC-MS/MS Data Analysis

PEAKS Studio 12 XPRO software (Bioinformatic Solutions) was used for analysing MS data; the PEAKS algorithm parameters were as follows: 20 ppm precursor and 0.1 Da fragment mass tolerance, methionine oxidation and cysteinylation as variable modifications having a maximum of two variable modifications, and 1–6 precursor charge. Default PEAKS algorithm search parameters were 15 de novo score (%) threshold and 30.0 peptide hit threshold (−10 logP). The search was complemented by the SPIDER algorithm to allow for single mutations. Peptides were filtered with 1% FDR; de novo only score was >=80% (see Supplementary material S1).

Unique self-peptide and bacterial sequences of more than 9 amino acids (aa)-long from each individual were selected from the immunopeptidomics results for compiling the database; peptides having post-translational modifications were excluded. Immunopeptidomics analysis considered previously reported aspects, such as peptide length distribution, peptide motif analysis, peptide clustering based on shared sequence features, and peptide SLA-binding prediction [40,41].

### 2.7. Immunoinformatics Analysis

The GibbsCluster 2.0 server (https://services.healthtech.dtu.dk/services/GibbsCluster-2.0/; accessed on 8 January 2024) for unsupervised alignment and clustering of peptide sequences was used for identifying MHC II elution ligand binding motifs obtained by spectrometry (MS). Data regarding self-peptides and bacteria were size-filtered, from 9 to 50 aa long; previously reported parameters for class II molecules were used in each experiment [41]. Briefly, the peptide sequences were loaded from the GibbsCluster 2.0 server using Gibbs clustering algorithm parameters; the number of iterations per sequence per temperature step was 20, using 10 temperature steps. The trash cluster option was activated. The core results from this analysis were used for grouping the peptides for each dataset based on shared sequence characteristics [40].

The data were analysed considering each pig’s haplotype obtained by genotyping for identifying the eluted ligands in silico. Unique peptides with greater than 9 aa in length that were obtained in each experiment were used in such analysis; predicting DRB1 alleles obtained by genotyping involved combining them with the 14 DRA alleles from the IPD-MHC database (https://www.ebi.ac.uk/ipd/mhc/; accessed on 26 March 2022). DQB1 gene PCR genotyping results were considered for assigning DQA gene haplotypes. DQA gene haplotype reports in the pertinent literature were reviewed; however, due to a lack of pertinent genotyping data, the allele obtained by genotyping was considered for predicting DQB1 and estimating the DQA chain by combinatorics because one was dealing with low-resolution genotyping.

The peptide sequences were used for analysing SLA binding prediction using the MixMHC2pred predictor (http://mixmhc2pred.gfellerlab.org/; accessed on 16 July 2024). This involved using the haplotypes of the individuals sampled in the study, i.e., peptides with 12 to 21 aa length were considered, and peptides having %Rank values close to zero were considered the best.

NetMHCIIpan-4.0 [20], which uses artificial neural networks (ANNs), was used for predicting the assigned alleles. NetMHCIIpan-4.0 predicted values for a peptide/receptor system are given as an elution score and an IC_50_ value for affinity and may include information regarding a target ligand’s proteolytic context from a particular sequence being analysed.

Although the aforementioned network version does not include pig MHC II alleles, the alpha- and beta-chain sequences of the alleles of interest can be introduced as a pseudo-sequence, i.e., a set of positions for both the alpha (13 aa) and beta chains (21 aa) constituting residues potentially in contact with a target peptide in the MHC–peptide complex [42].

The predicted NetMHCIIpan-4.0 scores may vary from receptor to receptor; a %Rank value is estimated as a prediction score to make unbiased comparisons, where the lower the %Rank, the greater the receptor binding. The purpose of such a %Rank value is to compare the peptide in question with a possible universe of random peptides. The following %Rank values were used for classifying the target peptides: less than 5% was considered weak binding, and less than 1% was considered strong binding.

NetMHCIIpan-4.0 does not provide %Rank values as it does not include pig MHCII alleles; thus, the %Rank distribution of elution and the affinity of a random set of peptides for each allele was calculated for estimating it, using a set of 1,800,000 non-redundant peptides derived from UnitPro-tKB/Swiss-Prot UniProt [43] (200,000 peptides for each 13 to 21 aa length). It was thus possible to estimate whether a peptide from a new pseudo-sequence was a weak-binding or a strong-binding peptide.

Seq2Logo was used for creating elution and binding affinity logos for each allele based on strong binding peptides’ 9 aa binding core (range <1 percentile) using the Kullback–Leibler quantile approach [44].

## 3. Results

### 3.1. Haplotype Identification

The three homozygous individuals had 100% BLAST identity with the SLA-DQB1 sequences reported in the IPD-MHC database. SLA-DRB1 [35] and SLA-DQB1 genotyping results were used for assigning the SLA-DQA genes, based on previous haplotype reports [45]. It was observed that each pig had a different haplotype (Table 1).

### 3.2. SLA Class II Ligand Ligandome Data Analysis and Binding Affinity

#### 3.2.1. Peptide Length Distribution

**Figure 1:**
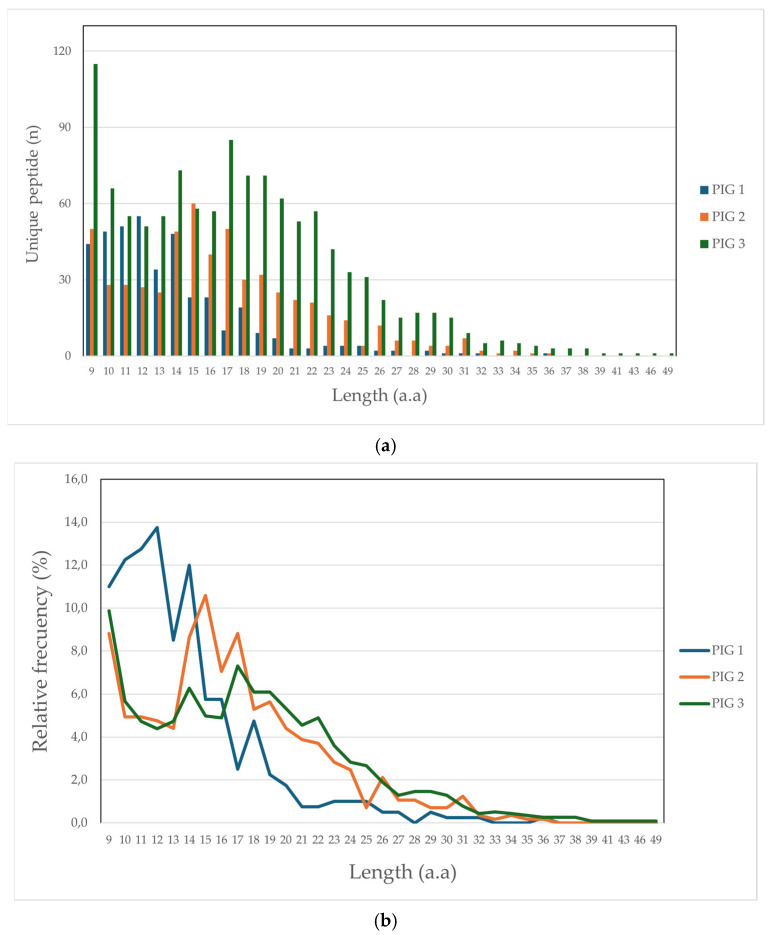
Comparative peptide length distribution: (**a**) frequency peptide; (**b**) peptide percentage.

Altogether, 1990 unique peptides were identified (1.9% being peptides derived from bacterial presentation), having less than 10 ppm, 1% FDR, and being more than 9 aa in length, distributed as follows: 372 for Pig 1 (Lr-0.23), 438 for Pig 2 (Lr.0.12) and 1180 for Pig 3 (Lr.0.21). It was observed that peptide length obtained in this study was up to 36 aa for Pigs 1 and 2 and 49 aa for Pig 3. Figure 1 illustrates peptide frequency (a) and proportion (b); it was found that peptides having 9, 14, and 17 aa lengths had greater representation concerning both parameters, mainly regarding Pigs 2 and 3. Greater frequency and proportion were found for Pig 1, peptides having lengths between 10 and 14 aa.

#### 3.2.2. Motif Deconvolution

**Figure 2:**
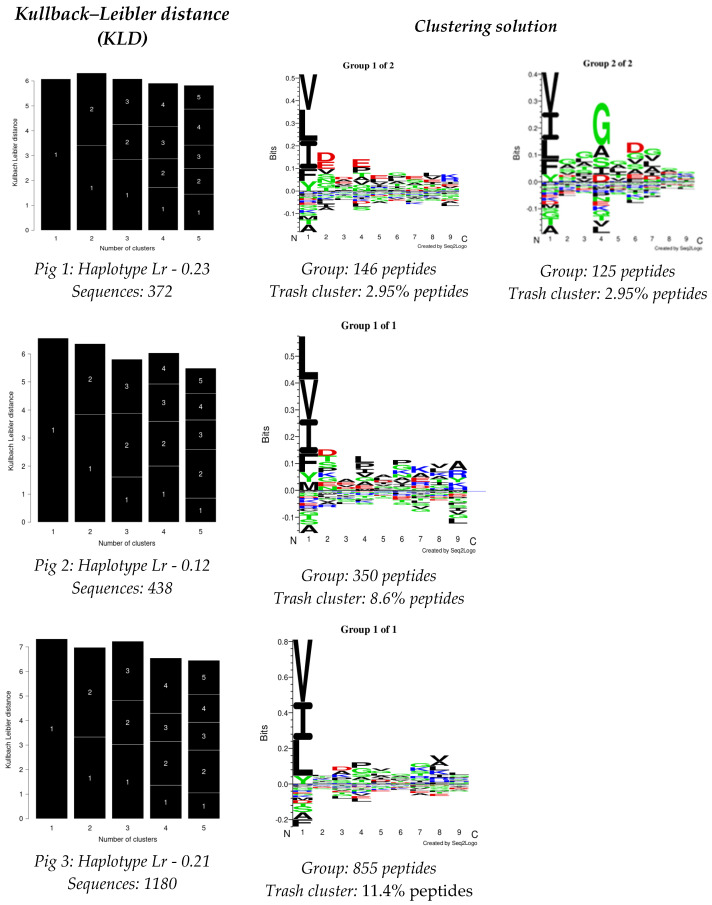
GibbsCluster 2.0 results for the dataset for peptides obtained per pig in this study. Sequences: the number of peptides used for each analysis. Group: the number of peptides selected by the server for creating elution motifs. Trash cluster: the percentage of peptides removed as being outliers, considering a 2 threshold.

The GibbsCluster 2.0 server was used for analysing the data obtained from each experiment; the deconvolution motifs were analysed as mentioned above. The highest Kullback–Leibler distance (KLD) was considered for selecting the best solution, represented by the GibbsCluster server’s Seq2Logo. Two solutions were observed for Pig 1, and a single solution was found for the other two pigs in the study (Figure 2). Regarding Pig 1, different aa preferences were identified in anchor positions P1, P4, and P6 in both solutions, and additional differences were also found in P2 and P7. Pig 2 data revealed aa preferences in positions P1, P4, P6, and P9, accompanied in turn by preferences in positions P2, P7, and P8. Notably, aa preferences stood out in anchor positions P1 and P4 for Pig 3, while preferences were also observed for positions P3, P7, and P8.

#### 3.2.3. Peptide Clustering Based on Shared Sequence Features

Motifs were deconvoluted using GibbsCluster 2.0 server to obtain data on the peptides’ best solutions; peptide binding regions (COREs) (organised as an example in Table 2) were identified considering the parameters described in the Section 2.7 Immunoinformatics Analysis, and the results for Pig 1 (Lr-0.23) are presented (see Supplementary Material S2, Table S22, for more results).

#### 3.2.4. SLA-Binding Prediction: Deconvolution Motif and Annotation

The MixMHC2pred MHC II ligand predictor gave few binders in the three datasets analysed; thus, 12 to 21 aa long peptides were used for this purpose. A %Rank value lower than 1% was observed for Pig 1 in 2.26% (5/221) of the peptides analysed, where two peptides had strong binding to SLA-DR alleles and the remainder to SLA-DQ alleles. This binding pattern was similar for Pigs 2 and 3, where it was observed that 3.92% (8/204) and 2.25% (9/400) of the peptides analysed had less than 1%Rank value. Regarding allele binding preferences, it was observed for Pig 2 that preference was similar for both alleles: four for DR and four for DQ. For the Pig 3 binding preference was observed for DR alleles in six out of nine peptides, the other three peptides were observed for DQ.

**Figure 3:**
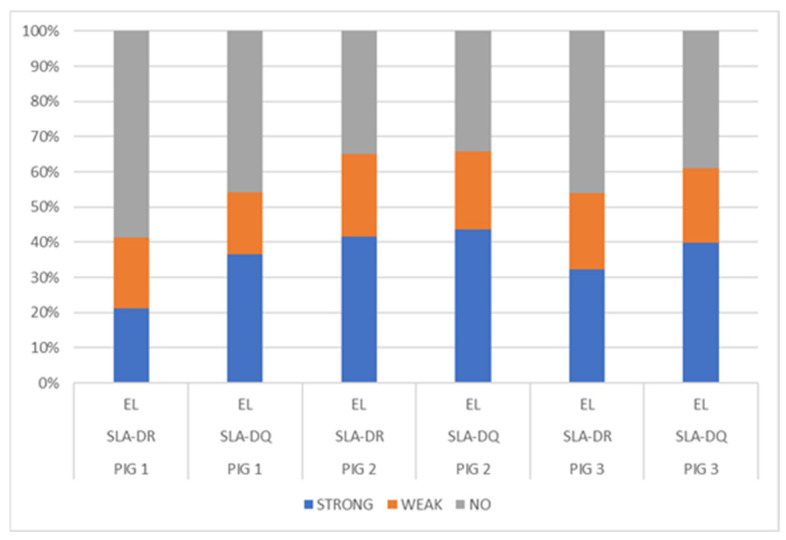
Distribution of eluted ligand (EL) prediction, considering total data percentages: strong EL (<1%Rank), weak EL (<5%Rank), and no EL (≥5%Rank).

NetMHCIIpan-4.0, using artificial neural networks (ANNs), predicted a greater number of eluted ligands for MHC II, SLA-DR, and SLA-DQ molecules (organised in Table 3), mainly for Pig 2 having haplotype Lr.0.12. In turn, the predicted elution ligand percentage results were compared for each dataset analysed, considering SLA-DR and SLA-DQ (Figure 3); a fair percentage of elution was predicted for the peptides analysed in the three pigs, particularly peptides from Pig 2 (haplotype Lr-0.12), which had a greater percentage of eluted ligands (<1%) compared to that for the other two pigs. A higher %prediction of ligands eluted by SLA-DQ was observed in the three pigs, and by SLA-DR to a lesser extent, mainly in Pig 1 (haplotype Lr-0.23). Class II molecule binding was predicted, observing that the Pig 2 haplotype (Lr-0.12) had the greatest number of peptides having strong binding (<1%) (Table S23 and Figure S19).

**Figure 4:**
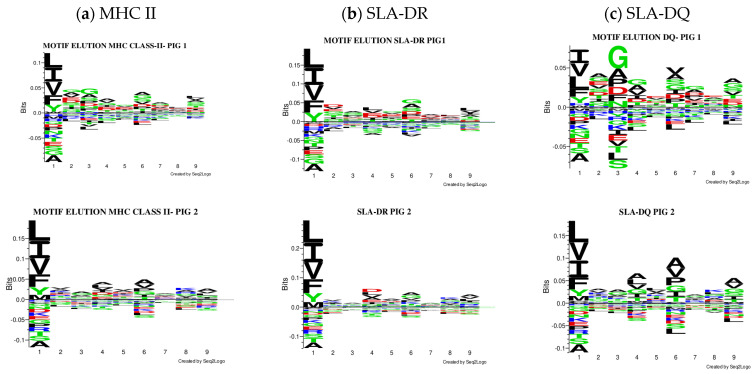

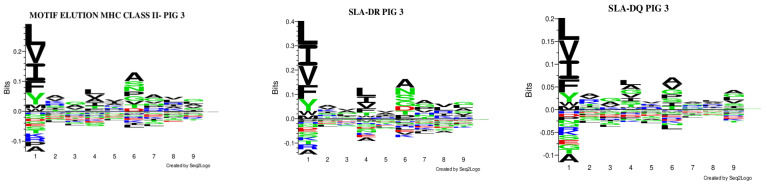
Comparison of the logos obtained, considering only peptides predicted to be eluted ligands (EL < 5%Rank). All the results were considered for creating the MHC II (**a**), SLA-DR (**b**), and SLA-DQ (**c**) logos for each pig.

The Seq2Logo server predicted logos for peptides having the best elution percentages (organised in Figure 4). Each MHC II molecule’s contribution was predicted, considering eluted ligands having less than 5% value in all the pigs analysed. Similarly, affinity logos were predicted, and a similar pattern was observed (Figure S20).

**Figure 5:**
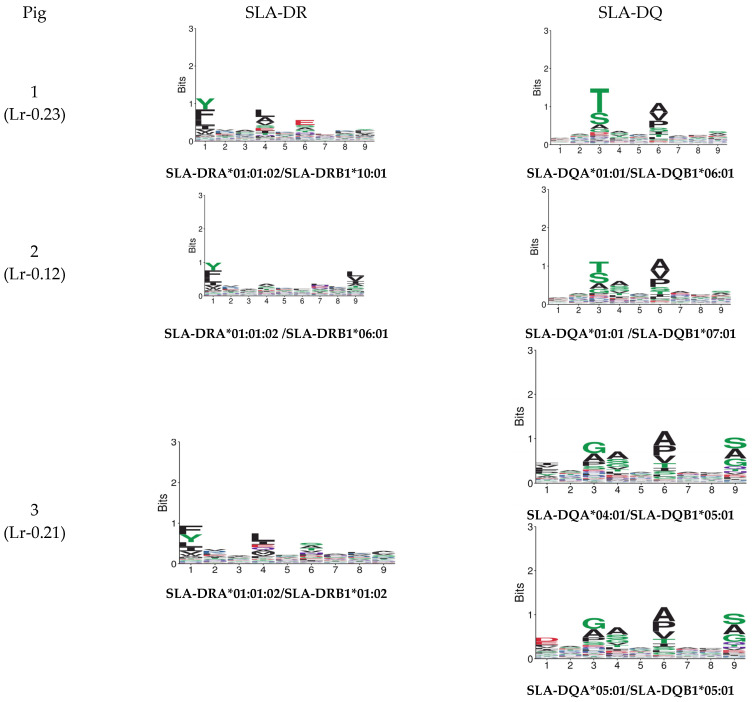
A MixMHC2pred motif was predicted from the haplotype sequences for the pigs used in this study.

**Figure 6:**
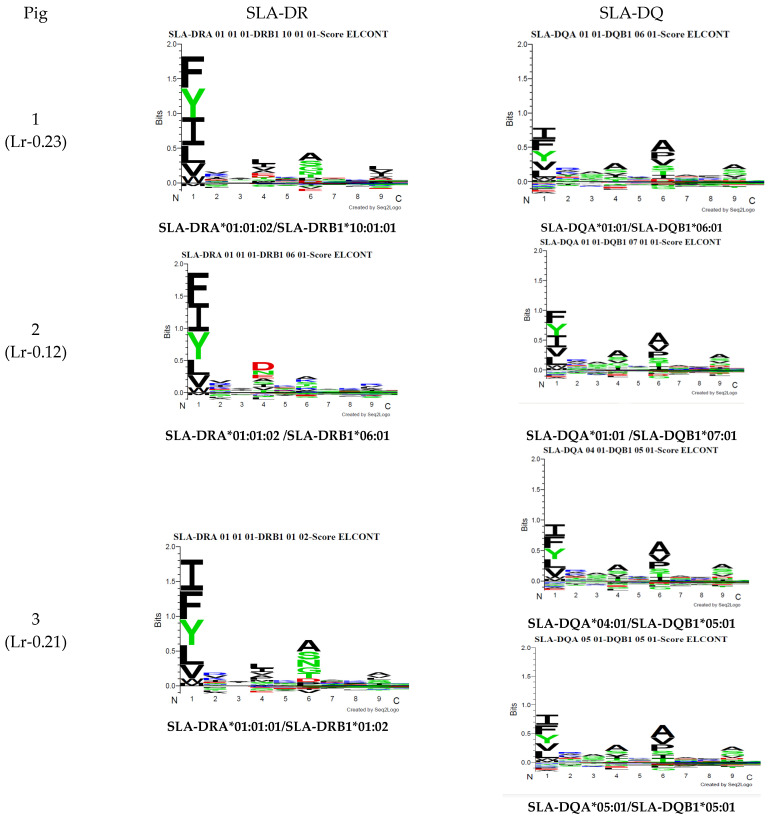
A NetMHCIIpan-4.0 motif was predicted from the haplotype sequences from the pigs used in this study.

Figure 5 gives MixMHC2Pred binding specificity results for both predictors. Figure 6 gives NetMHCIIpan-4.0 results. A single combination was selected regarding DRA and DQA chain combinations for illustrative purposes (data available upon request). There were noticeable differences regarding peptide anchoring positions, especially concerning MixMHC2Pred server DQ predictions.

### 3.3. Salmonella typhimurium Infection Related Peptidome

**Figure 7:**
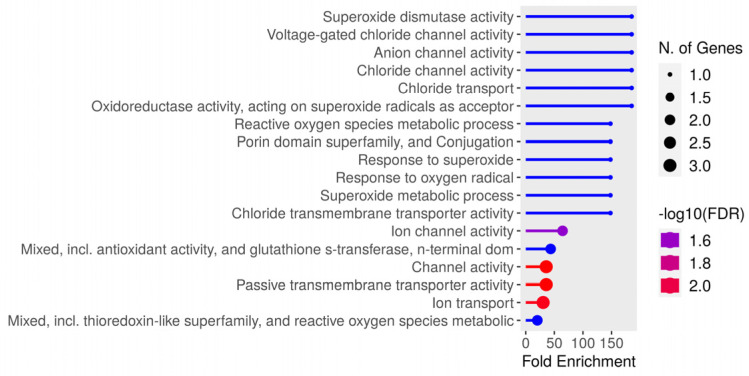
Distribution of Salmonella enterica gene, serovar Typhimurium enrichment identified in this study.

Thirty-one unique peptides were identified from the bacteria. Greater enrichment was observed regarding genes related to superoxide dismutase (SOD) activity, voltage, anion, and chlorine channels in enrichment analysis. The identified gene distribution is represented in Figure 7, while Table 4 lists the peptide sequences and protein identification, where it can be observed that there was more than one peptide in several proteins. The NetMHCIIpan-4.0 neural network identified eluted bacterial peptides; the greatest number of identifications were for Pig 3 (<5%Rank) for both DR (13 peptides) and DQ (16 peptides); most peptides had >1%Rank (Strong), and 87% (27/31) of the unique peptides identified had <5%Rank elution (highlighted in bold in Table 4).

## 4. Discussion

*Salmonella* spp. is a pathogen that is responsible for several million cases of typhoid fever and non-typhoid salmonellosis in humans annually [47]. New vaccination alternatives are required for promoting salmonellosis prevention and control in swine, mainly due to its impact on human health [10]. Immunopeptidomics for MHC class II immunocomplexes is a key tool for identifying epitopes regarding the rational development of vaccines in pigs. The impact of pig breed on macrophage response to lipopolysaccharides and the compartmental tissue from which they were obtained has been evaluated, finding that peripheral blood monocyte-derived macrophages (PBMMs) overexpress class II molecules [48]. Here, pig macrophages genotyped for class II molecules, derived from peripheral blood monocytes and stimulated with *Salmonella enterica* serovar *Typhimurium*, were used as the source of analysis (1:40 MOI for 20 h).

Similar to experiments involving humans, immunopurification has been aimed at capturing immune complexes from DR molecules [49]. The L243 monoclonal antibody has been used, which has already been shown to cross-react with the target species [39]. Regarding self-peptide percentage versus that for bacteria, as in other species, the number of self-peptides eluted was significantly higher (98.4%) than that for bacterial peptides [22].

Donor individuals were genotyped for SLA-DRB1 and SLA-DQB1 genes for a better understanding of the immunopeptidome characteristics of the species being studied as they are the most polymorphic [30] and are potentially coeluted [50]. Thus, the pigs’ haplotypes were assigned via SLA-DQB1 gene genotyping information, which enabled adjusting the NetMHCII-pan-4.0 neural network and analysing the performance of other currently available prediction methods (such as MixMHC2pred).

High-resolution PCR used for SLA-DRB1 [35] and SLA-DQB1 genes led to the identification of three of the haplotypes reported for class II gene species [51,52]. Previous data concerning haplotype frequency in German purebred Landrace pigs were 10% for haplotype Lr-0.23 (Pig 1), 40.8% for Lr-0.12 (Pig 2), and 12% for Lr-0.21 (Pig 3) [45]. A study by Hammer et al. (2021) used low-resolution genotyping of European commercial pig populations, and 12.46% haplotype frequency was reported for Lr-0.12, 11.58% for Lr-0.23, and 6.01% for Lr-0.21. It was concluded that haplotype SLA-II Lr-0.12 was one of the most abundant in European pig production [52]. Remarkably, haplotypes Lr-0.23 and Lr.0.12 found in this study coincided with data concerning the most abundant haplotypes reported in the pertinent literature.

Peptide length plays a crucial role in peptide vaccine development as it directly influences a peptide’s MHC molecule binding affinity and its ability to be recognised by T cells. Optimal peptide length ensures a proper fit within the MHC binding groove, thereby facilitating stable presentation on the cell surface and enhancing T-cell receptor engagement. Shorter or longer peptides may not bind effectively, thus reducing immunogenicity and limiting vaccine efficacy. Appropriate peptide length is essential for eliciting specific immune responses and thereby avoiding cross-reactivity and minimising potential side effects [53]. Peptide length was more heterogeneous due to proteolytic pathway variation in class II molecules [54]. Peptides presented by class II molecules, which are loaded into the peptide-binding region for presentation on the cell surface, typically have 12 to 25 aa length [55].

In this study, it was observed that the peptide length distribution was consistent amongst datasets but varied with each haplotype analysed. Peptides 9 to 21 aa in length had the highest presentation frequency, as reported in previous studies on MHC II molecules in other species [56].

The peak for 9–11 aa long peptides observed in the three datasets may have been due to SLA-I co-purification during the experimental procedure. Class I molecule genotyping data from previous studies in bovines has been based on using neural networks for predicting class I ligands to differentiate this effect, along with immunopurification for class I molecules prior to processing class II molecules [22]. None of these processes could be carried out in this study due to the lack of genotyping information regarding the target population and specific antibodies for class I molecules. However, a low detection rate of strong MHC I binder peptides was observed when using NetMHCpan-4.1 (https://services.healthtech.dtu.dk/services/NetMHCpan-4.1/; accessed on 4 August 2024) for prediction with a set of representative alleles available for pig class I molecules. Some peptide overlapping was observed in the three datasets, characteristic of enzyme processing regarding class II molecule presentation.

The Cluster predictor was relied on in this study for its ability to remove contaminating residues after FDR filtration; it can deconvolute multiple motifs in a mixture of variable length peptides, thereby being useful for class I and II molecules in an unsupervised manner with alleles lacking previous reports [57]. Differences were observed between each pig’s deconvolution motif results obtained from the immunopeptidomics data. The SLA-II motifs identified in samples having SLA-II alleles were similar to most anchor positions reported for other species [58]. Differences were observed regarding the dataset analysed for each pig; thus, a single cluster was identified as the best solution for pigs 2 and 3, representing the pattern of the data analysed. However, two possible solutions were found for Pig 1, representing the data pattern, despite the fact that all individuals were homozygous. This differs from previous reports concerning monoallelic cells [27], which may indicate coelution with other MHC molecules (class I and SLA-DQ), thus increasing the peptide background and therefore hampering finding a single solution logo.

Variation regarding the analysed position specificities was also identified; the GibbsCluster predictor cannot directly assign each ligand’s MHC restriction since this requires comparing unsupervised motifs with the published binding motifs for the MHC molecules in a particular sample [59]. This approach is comparative regarding most human alleles, the most prevalent of which have been well characterised and documented [40]. The data obtained in this study should be complemented in the future with a larger number of studies for further characterising pig class II molecules’ immunopeptidome.

Limited peptide coverage in this study should be acknowledged as a potential limitation that could affect the comprehensiveness and reliability of the conclusions. Insufficient peptide representation may result in the incomplete characterisation of the immunopeptidome, potentially missing key epitopes that may be involved in effective immune responses. This issue is particularly significant when attempting to generalise findings regarding broader vaccine development or immunological research. A narrow peptide dataset may fail to capture the full diversity of the antigen presentation repertoire, thus potentially biasing the study of immunological outcomes. Future studies must incorporate broader peptide sampling and more extensive mass spectrometry analysis to improve result reliability. Such an approach would enable a more comprehensive identification of relevant epitopes, thereby enhancing these findings’ translational applicability for vaccine development.

The coverage of available experimental data concerning MHC II alleles is limited. Experimentally determining binding motifs in humans, and even more so in animals, has promoted the development of ligand prediction tools, even for alleles for which no data have been reported so far [20]. This study involved using two neural networks for predicting SLA class II binding specificity from amino acid sequences obtained in immunopeptidomics assays. A few small peptides having pig MHC-II binding preference were observed when analysing the dataset obtained for the MixMHC2pred interface; a greater number of mass spectrometry data may thus be required for improving the model’s predictive capability [40].

The number of peptides predicted to be eluted ligands was better for both molecules with the NetMHCIIpan-4.0 neural network tuned with the pig alleles identified in this study. However, differences between individuals were observed as the number of peptides predicted for Pig 1 (Lr-0.23) was lower than for the other two. Various factors could have affected class II molecule affinity prediction capability, including the nature of MHC-II molecules, variation in peptide length, flanking regions’ influence, and correct identification of the core in the peptide binding region [60]. Demmers et al. (2021) tried to clarify mechanistic and adaptive differences between HLA systems; they challenged the B-lymphoblastic cell line (JY) with a high-temperature treatment imitating a “febrile state”, observing that high temperature can prepare these cells for an immune response having a greater capacity for HLA class II presentation and specific CLIP peptide release from the invariant chain [61].

Differences became evident when analysing the logos from peptides having the best prediction values as elution ligands. The overall elution preferences (MHC class II) were only visualised for DR and DQ when separating the logos for each molecule. Although the immunopurification method targeted SLA-DR molecules, the results suggested the presence of peptides presented by SLA-DQ molecules; however, this was not confirmed experimentally. Most human immunopeptidomics data have centred on human leukocyte antigen (HLA)-DR, and to date, few immunopeptidomics data exist that deal with HLA-DQ [49]. The use of pan-HLA class II antibodies has shown low specificity against DQ and DP, leading to a low peptide yield for these loci [62]. Nilsonn et al. (2023) developed a method for accurately predicting HLA class II antigen presentation; this involved updating the NetMHCIIpan-4.0 prediction method for working with three loci, using immunopeptidomics data, and machine learning.

Variations were observed in both neural networks when predicting binding specificities. The MixMHC2pred interface can obtain MHC-II motifs for any MHC-II allele, considering alpha- and beta-chain amino acids [20]. All haplotype combinations were thus selected, considering alpha alleles reported to date as such information can lead to predicting the presentation of MHC class II ligands and CD4+ T-cell epitopes (http://mixmhc2pred.gfellerlab.org/; accessed on 21 July 2024). The SLA-DR allele results coincided regarding binding specificities similar to that for other species; however, for SLA-DQ, differences were observed concerning binding specificities in the peptides’ preferred anchoring positions (especially in Pocket 3).

Racle et al. (2019) analysed 40,864 unique ligands from 13 HLA-II cell lines and/or tissue samples and combined them with laboratory-derived samples. They analysed 77,189 unique peptides from 23 samples, which led to the development of a deconvolution motif algorithm/software (MoDec). Various motifs were found that were extremely similar to alleles, mainly HLA-DR, but also for HLA-DP and HLA-DQ. This study showed that the motif reported for HLA-DQA1*02:01/DQB1*02:02 using MixMHC2pred had preferences similar to those found in our study, specifically in CM647 and GD149 cells’ Pocket 3. Racle’s study also analysed NetMHCIIpan-4.0 motifs, observing preferences in normally known pockets, which coincided with the data produced from this study [58].

Most data on motifs have been produced regarding humans; there are few reports concerning other species, i.e., reports of four alleles in mice, seven alleles in cattle, and two alleles in birds [20] (www.mhcmotifatlas.org/; accessed on 16 July 2024). No information was found regarding motifs in the species analysed in this study at the time of writing this manuscript.

Regarding bacterial peptides, it was observed that 28 aa long peptides had <5% Rank value concerning their elution and binding to MHC, using the NetMHCIIpan-4.0 neural network; this feature could not be determined using the MixMHC2pred predictor since it only allows for the analysis of 12 to 21 aa long peptides. There is clearly a need for obtaining more immunopeptidomics analysis data for improving predictor performance [63].

Bacterial peptides were successfully identified in this study, thereby confirming immunopeptidomics’ usefulness for identifying epitopes. Such presentation enables CD4+ T-helper cell activation which, in turn, could stimulate various immune responses, including B-cell activation for producing specific antibodies and cytotoxic CD8+ T-cell recruitment. Regarding *Salmonella* infection, these peptides can prime the immune system to recognise and respond more rapidly to the pathogen, thereby enhancing pathogen clearance and conferring protection. Effective peptide vaccines could mimic such natural processes, promoting robust and targeted immunity against Salmonella and reducing infection severity [64].

Overall, 31 unique bacterial peptides were found, 5 of which belonged to outer membrane proteins, and 27 peptides had good correlation with elution prediction when the NetMHCIIpan-4.0 adjusted neural network was used for analysing them, and only 3 had been previously reported by the group [39]. Karunakaran et al. (2017) identified 87 class II peptides via C57BL/6 mouse bone marrow-derived dendritic cell assays. The cells had been infected by *Salmonella enterica* strain SL1344, having different MOI for 14 h, although none of them coincided with those reported in this study.

Extracellular proteins or those on bacterial cell surfaces are considered good candidates for vaccines, due to their access to host immune systems [65]. The OmpA outer membrane protein family is a group of porin proteins that are exposed on the cell surface and are mainly present in Gram-negative bacteria. They are related to bacterial adhesion, invasion, and/or intracellular survival, evading host response or proinflammatory cytokine production, which has led to them being evaluated as possible vaccine candidates [66]. Five peptides belonging to this protein were identified in this study. Intracellular chaperone proteins such as GroEL were initially associated with protein folding; a study by Fourie et al. (2020) highlighted the fact that these proteins become relocated to the cell surface or secreted during invasion, thereby enabling them to be recognised as antigens by the immune system [67]. The present study found 10 unique peptides. All the peptides identified in the two aforementioned proteins can be considered possible candidates for developing a multi-epitope vaccine contributing to salmonellosis control.

MHC-associated peptide proteomics (immunopeptidomics) is used to directly identify the peptides derived from a specific protein/antigen and is the only method providing direct evidence of actionable and cell surface presented epitopes [68]. These are potential T-cell epitopes since such assays require MHC protein proteolytic processing and antigen/peptide presentation by the immune system; they are thus used for studying protein immunogenicity [69]. Moreover, immunopeptidomics-confirmed T-cell epitopes are known to be immunogenic in tumour-related settings, but their validation could be challenging.

Human MHC molecules’ binding specificities have been characterised using in vitro assays; however, full characterisation is impossible due to large polymorphism (specifically in the peptide binding region) and the number of allele variants per locus. Mass spectrometry techniques’ potential is based on the number of data that can be detected and antigen presentation properties, including binding affinity, binding stability, processing, translocation, and chaperone action on binding [40,70]. More than one million natural ligands had been identified in different tissues, treatments, and species by 2022. Such data have enabled improving the ability to predict ligands naturally presented by MHC molecules and T-cell epitopes in humans and other organisms [24].

Several in vitro immunogenicity tests could be used for experimentally testing identified peptides’ in vitro proof of immunogenicity (i.e., T-cell reactivity) [68,71]. Synthetic peptides are one of the first in vitro options for validating MS data, though plasmids encoding recombinant proteins could be produced for transfecting pig macrophages or other cell lines and evaluate key peptide/epitope processing and presentation by immunopeptidomics. Also, immunogenicity screenings could be performed with individual epitopes in vitro [72]. All the forgoing highlights the complexity of immunological validation -T-cell recognition- and immunopeptidomics’ power and feasibility for next-generation bacterial vaccine development [73].

Further studies are required for evaluating/ascertaining the immunogenic capacity of the peptides reported in this study; this would enable the creation of a peptide subunit-based vaccine as an alternative for controlling porcine salmonellosis. Immunoinformatics studies the relationship between immune response and predicted epitopes. This has led to the development of a variety of tools for B- and T-epitope prediction. It represents a simple methodology and does not require complex skills, and most tools are free to use. The main disadvantage is that all computational prediction tools are based on computational approximations involving chemistry and biology, and they never achieve 100% accuracy [74].

The immunogenic potential of the identified peptides could be experimentally tested in vivo in *S. thyphimurium*-infected animals [75]. Different constructs could be designed for obtaining a multi-epitope vaccine; this would involve peptides being connected to linker sequences to maximise processing and immunogenicity [76] and, later, in animal models by different vaccine formulations to demonstrate protective immunity [77].

Moise et al. (2020) have recently reported the use of the PigMatrix algorithm for *predicting* SLA class II T cell epitopes [78] and the EpiCC online tool for predicting PCV2 vaccine efficacy for identifying T-epitopes and analysing vaccine efficacy. These were integrated into an iVAX platform for designing epitope-driven vaccines for humans. Despite the progress made and reported, the authors recommended identifying the alleles most represented in worldwide populations. They also mentioned that more extensive data on SLA-restricted peptides are needed for further evaluating the PigMatrix approach and improving its predictive capacity via binding assays involving commonly expressed SLA molecules [63]. Data obtained from immunopeptidomics analysis thus prove beneficial for improving predictor capability.

We have thus provided experimental evidence involving MHC-associated peptide proteomic assays of antigen-presenting cells regarding (i) the number of unique infection-derived peptides isolated and such peptides’ average length; (ii) the derived peptides for each genotyped subject and motifs identified, showing that affinities for MHC-II specific variants varied between individuals; (iii) *S. thypimurium* pathogen-specific peptides; and (iv) binding specificities or in silico binding predictions for evaluating overall dataset quality.

## 5. Conclusions

Immunopeptidomics is very useful for identifying new bacterial antigens, thereby promoting the development of new-generation peptide subunit-based vaccines contributing to salmonellosis control. Thirty-one bacterial peptides were identified, with most being predicted by the neural network adapted for such purpose.

Our study has provided a detailed in vitro analysis of immunogenic peptides using immunopeptidomics and computational models. These foundational data are crucial for identifying potential vaccine candidates and understanding MHC–peptide interactions. The methodology offers insights into the antigenic landscape and the predicted immunogenic epitopes, which can guide future in vivo studies and vaccine development. While in vivo validation is necessary for confirming efficacy, the study’s findings significantly contribute to the initial stages of rational vaccine design and immunological research, offering a robust basis for subsequent experimental investigations.

More immunopeptidomics data are required to improve neural network performance. Further work should be carried out concerning the immunogenicity analysis of the peptides found in this study. T-cell presentation-related alleles must be genotyped for a better understanding of and benefit from information regarding the immunopeptidome and predicting T-cell epitopes. The lack of information regarding pigs related to identifying allele families having greater representation in the population has challenged T-cell epitope-related vaccine development.

Obtaining a highly protective multi-epitopic vaccine will require combining the epitopes here described with further immunopeptidomics studies carried out with macrophages from pigs with different haplotypes, trying to cover the most frequent MHC alleles in the porcine population.

## Figures and Tables

**Table 1:** Class II allele genotyping results.

Pig	SLA-DRB1 ^1^	SLA-DQB1 ^2^	SLA-DQA ^3^	Haplotype ^3^
1	10:01:01	06:01	01:XX	Lr-0.23
2	06:01	07:01	01:XX	Lr-0.12
3	01:02	05:01	04:XX/05:XX	Lr-0.21

^1^ Genotyped by Sanger sequencing of the PCR-amplified product (previously reported) [35]; ^2^ genotyped by Sanger sequencing of the PCR-amplified product in this study; ^3^ previously reported [45].

**Table 2:** Representation of the peptides obtained from Pig 1 (Lr-0.23), constructed from shared class II molecule sequences. The CORE (portion of a peptide in the alignment window) [46] is highlighted in bold in the peptide sequence, considering positions 1–9 identified in the elution motifs.

CORE Position
1 2 3 4 5 6 7 8 9
V**LSAADKANV**KAAWGKVGGQA
V**LSAADKANV**KAAWG
V**LSAADKANV**KAAWGKVGG
V**LSAADKANV**KAAWGKVGGQAGA
V**LSAADKANV**K
V**LSAADKANV**KAA
V**LSAADKANV**KAAW
V**LSAADKANV**KAAWGKVG
GSYTQAAGSDSAQGS**DVSLTKDPR**V
SYTQAAGSDSDQGS**DVSLTKDPR**V
GSDSAQGS**DVSLTKDPR**V
AGSDSAQGS**DVSLTKDPR**V
SDSAQGS**DVSLTKDPR**V
SYTQAAGSDSAQGS**DVSLTKDPR**V
S**DVSLTKDPR**V
SAQGS**DVSLTKDPR**V
GS**DVSLTKDPR**V
TED**LSSGLGVTK**QDL
TED**LSSGLGVTK**Q
TED**LSSGLGVTK**
TED**LSSGLGVTK**QD

**Table 3:** NetMHCIIpan-4.0 prediction for eluted ligands, considering the haplotype of the pigs used in this study.

ID	Eluted Ligand < 5%			Unique Eluted Ligand < 5%	
	Class II	SLA-DR	SLA-DQ	SLA-DR	SLA-DQ
Pig 1 (Lr-0.23)	3962	2149	1813	160	206
Pig 2 (Lr-0.12)	11764	3991	5343	287	202
Pig 3 (Lr-0.21)	3420	2511	3630	94	493

**Table 4:** Protein identification of Salmonella enterica, serovar Typhimurium MHC II bacterial peptides. The sequences having <5%Rank are shown in bold. Underlined peptides have been reported previously [39].

Protein Accession	Gene Name	Peptide Sequence	Total Peptides
P02936|OMPA_SALTY	Outer membrane protein A (outer membrane major heat-modifiable protein)	**APKDNTWYAGAKLGWSQYHDTGFIH**	2
		**GWSQYHDTG**	2
		**APKDNTWYAGAKLGWSQYHDTGFIHN**	1
		**RFGQQEAAPVVAPAPAPAPEVQ**	1
		**IGTRPDNGLLSVGVSYRFGQQEA**	1
P0A1D3|CH60_SALTY	Chaperonin GroEL (EC 5.6.1.7)	**VEDALHATRAAVEEGVVAGGGVALIRVASKIADL**	2
		**GNDARVKMLRGVNVLADAVKVTLGPKGR**	2
		**AAVEEGVVAGGGVALIRVASKIADL**	2
		**ATRAAVEEGVVAGGGVALIRVASKIADL**	2
		**AAVEEGVVAGGGVALIRVASKIADLKGQ**	1
P0A1H5|EFTU_SALTY	Elongation factor Tu (EF-Tu)	**GQVLAKPGTIKPH**	2
		**VDHGKTTLTAAITTVLAKTYGGAAR**	1
		**VNVGTIGHVDHGKTTLTA**	1
		**VDHGKTTLTAAITTVLAKTYGGAA**	1
P0A1P0|G3P_SALTY	Glyceraldehyde-3-phosphate dehydrogenase (GAPDH)	**VPTPNVSVVDLTVRLEKAATYEQIK**	1
		**DNETGYSNKVLDLIAHISK**	1
		**VPTPNVSVVDLTVRLEKAATYEQIKAAVK**	1
		**SNKVLDLIAHISK**	1
		**STGAAKAVGKVLPELNGKLTGMAF**	1
P0A2F4|SODF_SALTY	Superoxide dismutase [Fe]	SFELPALPY	1
P0A6B1|ACP_SALTY	Acyl carrier protein (ACP)	**ALEEEFDTEIPDEEAEKIT**	1
		**EEEFDTEIPDEEAEKITTVQ**	1
		**EEEFDTEIPDEEAEKIT**	1
P43019|SODM_SALTY	Superoxide dismutase [Mn]	SYTLPSLPY	1
Q7CPE2|ATPB_SALTY	ATP synthase subunit beta (EC 7.1.2.2) (ATP synthase F1 sector subunit beta)	**YTLAGTEVSALLGRMPSAV**	1
		**TLAGTEVSALLGRMPSAV**	1
		PADDLTDPSPA	1
		TLAGTEVSALL	1
Q8ZN40|ISCS_SALTY	Cysteine desulfurase (IscS)	**MKLPIYLDYSATTPVD**	1
Q8ZRP8|CLCA_SALTY	H(+)/Cl(-) exchange transporter ClcA	**GREGPTVQIGGNL**	1
Q8ZP45|Q8ZP45_SALTY	Aldehyde-alcohol dehydrogenase	**SVPETTKILIGEVTVVDESEPF**	1

## Data Availability

The logos of the alleles analysed in the present work with NETMHCIIPAN 4.0 are available upon request.

## Supplementary Materials

The following supporting information can be downloaded at: https://www.mdpi.com/article/10.3390/biology13100832/s1, Supplementary material S1—Protein identification summary by pig. Supplementary material S2.
