# Supplementary material for: Immunopeptidomics of Salmonella enterica Serovar Typhimurium-Infected Pig Macrophages Genotyped for Class II Molecules
Source: Biology (Basel). 2024 Oct 16;13(10):832. doi: 10.3390/biology13100832 (PMC11505383; doi:10.3390/biology13100832)
Supplement: Supplementary file 1 [file biology-13-00832-s001.zip › Supplementary Material S1. Protein Identification Summary by pig -1.pdf]

Protein Identification Summary-PIG 1

1. Notes

2. Result Statistics

Figure S1. False discovery rate (FDR) curve. X axis is the number of peptides being kept. Y axis is the corresponding FDR.

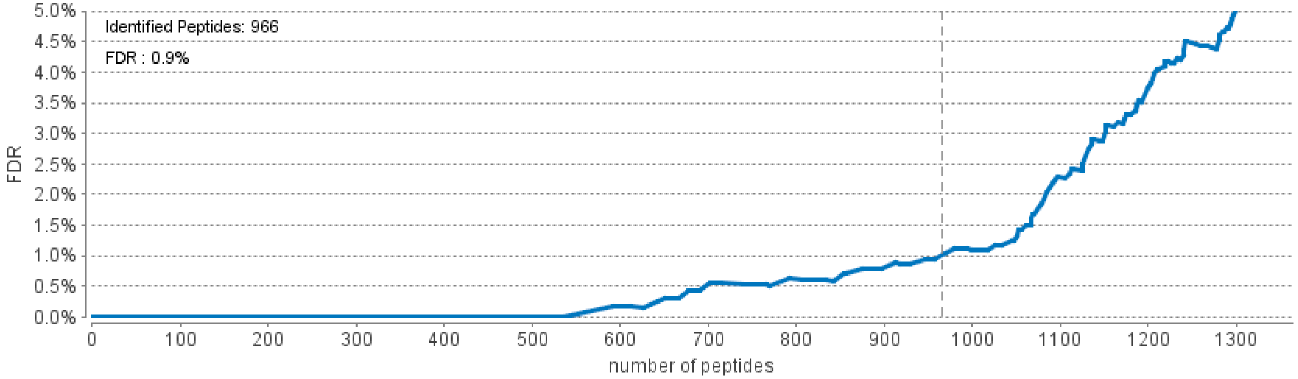

Figure S2. PSM score distribution. **(a)** Distribution of PEAKS peptide score; **(b)** Scatterplot of PEAKS peptide score versus precursor mass error.

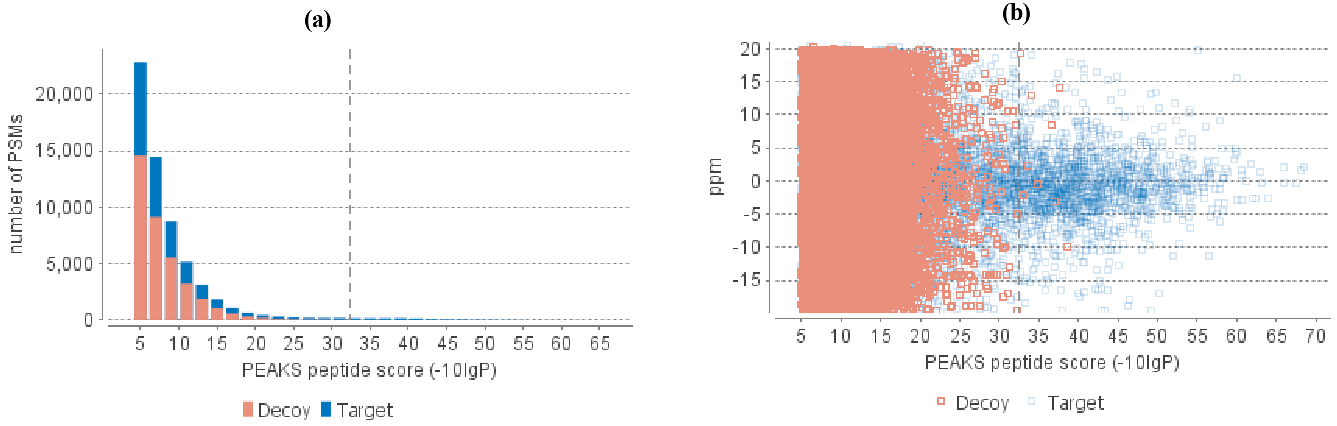

Table S1. Statistics of data.

	#Frames			#Features	#Precursors	Identified			#Peptides	#Sequences	#Proteins*		
	MS1	MS/MS	#Chimera			#PSMs	#Scans	#Features**			Groups	All	Top
Total	3787	37856	7137	25019	184029	1410	1410	552	967	938	169	640	431
0506_20_2_Fe_Terry_Salmonella	3787	37856	7137	25019	184029	1410	1410	552	967	938	169	640	431

* proteins with significant peptides are used in counts.
** features are identified by DB search only.

Figure S3. Sample overlap for Proteins and Peptides (up to 8 samples). **(a)** All Proteins; **(b)** Top Proteins; **(c)** Peptides;

(a)	(b)	(c)
**Not applicable to only one sample**	**Not applicable to only one sample**	**Not applicable to only one sample**

Figure S4. Distribution of peptide feature detection. **(a)** Feature m/z distribution; **(b)** Feature RT distribution.

(a)

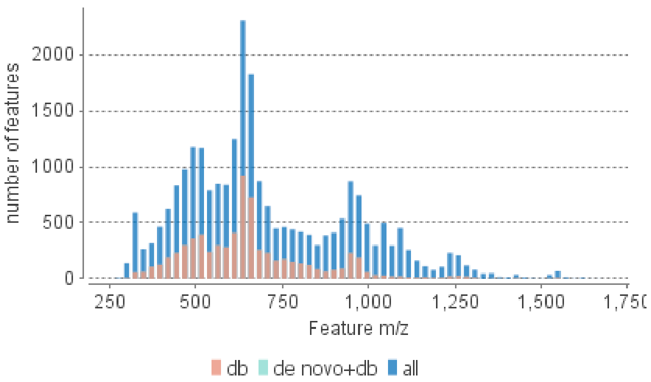

(b)

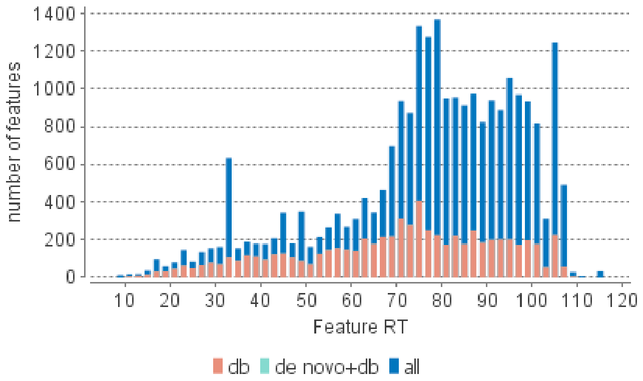

Figure S5. Distribution of identified peptide features. **(a)** Feature abundance distribution; **(b)** *De novo* sequencing validation.

(a)

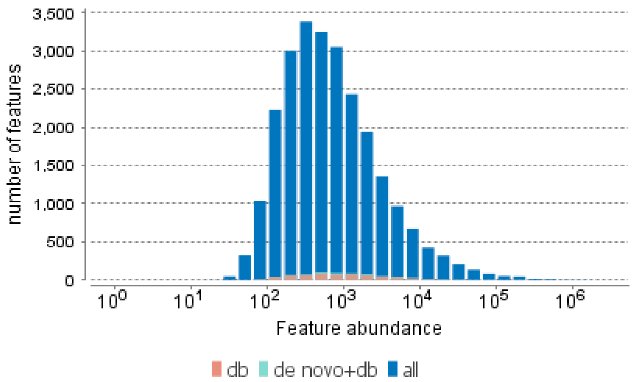

(b)

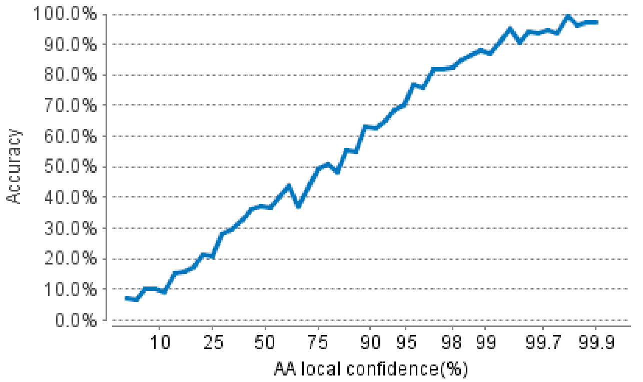

Table S2. Result filtration parameters.

Peptide -10lgP	≥32.4
PTM Ascore	≥95
Peptide mutation ion intensity	≥0%
Protein -10lgP	≥0

Table S4. PTM profile.

Name	ΔMass	Position	#PSM	-10lgP	Abundance	AScore
Oxidation	15.99	M	145	61.75		1000.00
Acetylation	42.01	N-term	59	62.98	1.85E3	1000.00

Proteins unique peptides	≥1
De novo score(%)	≥80%

Citrullination	.98	R	46	60.11	6.19E2	63.24
Deamidation	.98	NQ	33	60.11		1000.00

Table S3. Statistics of filtered result.

FDR (Peptide-Spectrum Matches)	0.6%
FDR (Peptide Sequences)	0.9%
FDR (Protein Group)	5.3%
De Novo Only Spectra	370

3. Experiment Control

Figure S6. Precursor mass error of peptide-spectrum matches (PSM) in filtered result. **(a)** Distribution of precursor mass error in ppm; **(b)** Scatterplot of precursor m/z versus precursor mass error in ppm.

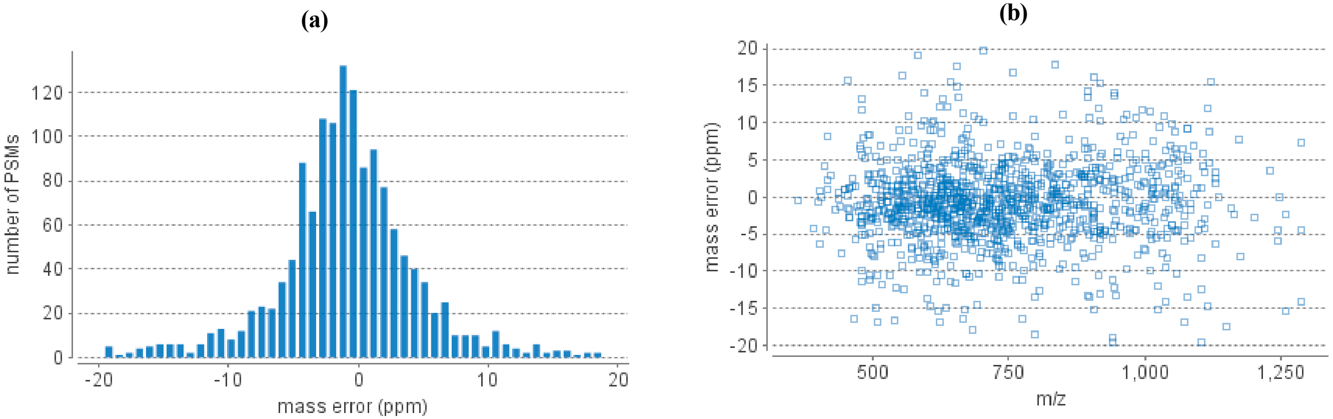

Table S5. Number of identified peptides in each sample by the number of missed cleavages.

Missed Cleavages	0	1	2	3	4+
0506_20_2_Fe_Terry_Salmonella	0	0	0	0	967

4. Other Information

Table S6. Search parameters.

Query Type: Homology Match
Variable Modifications:
  Oxidation (M): 15.99
  Cysteinylation: 119.00
Fragment ion tolerance: 0.1
L equals I: true
Q equals K: true
Report number: 1

Table S7. Instrument parameters.

Fractions: 0506_20_2_Salmonela_F2_Terry_Slot2-9_1_12536.d
Ion Source: ESI(nano-spray)
Fragmentation Mode: CID, CAD(y and b ions)
MS Scan Mode: TimsTOF
MS/MS Scan Mode: TimsTOF

Maximum # of PTMs: 2
De novo score(%) threshold: 15
Peptide hit threshold (-10logP): 30.0
Peaks run ID: 313
Merge Options: no merge
Precursor Options: corrected
Charge Options: no correction
Filter Charge: 1 - 6
Process: true
Associate chimera: yes

Protein Identification Summary-PIG 2

1. Notes

2. Result Statistics

Figure S7. False discovery rate (FDR) curve. X axis is the number of peptides being kept. Y axis is the corresponding FDR.

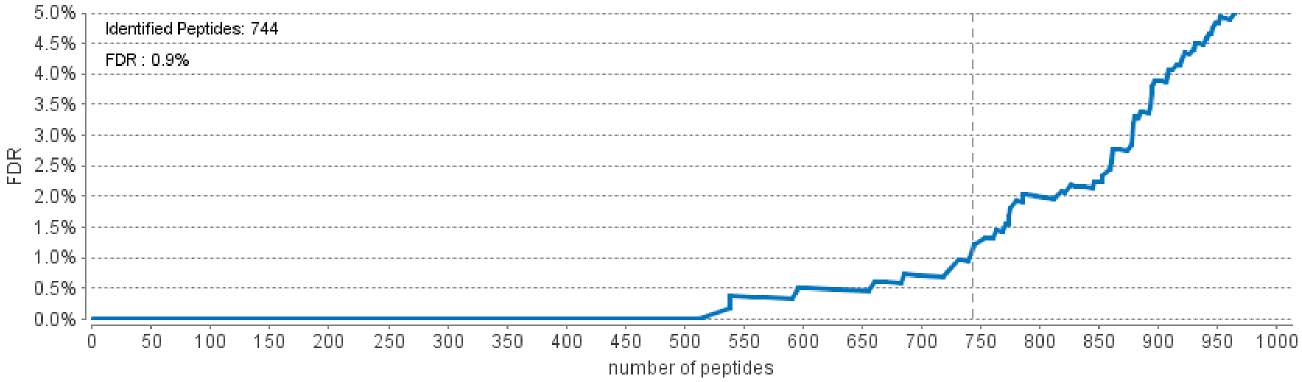

Figure S8. PSM score distribution. **(a)** Distribution of PEAKS peptide score; **(b)** Scatterplot of PEAKS peptide score versus precursor mass error.

(a) **(b)**

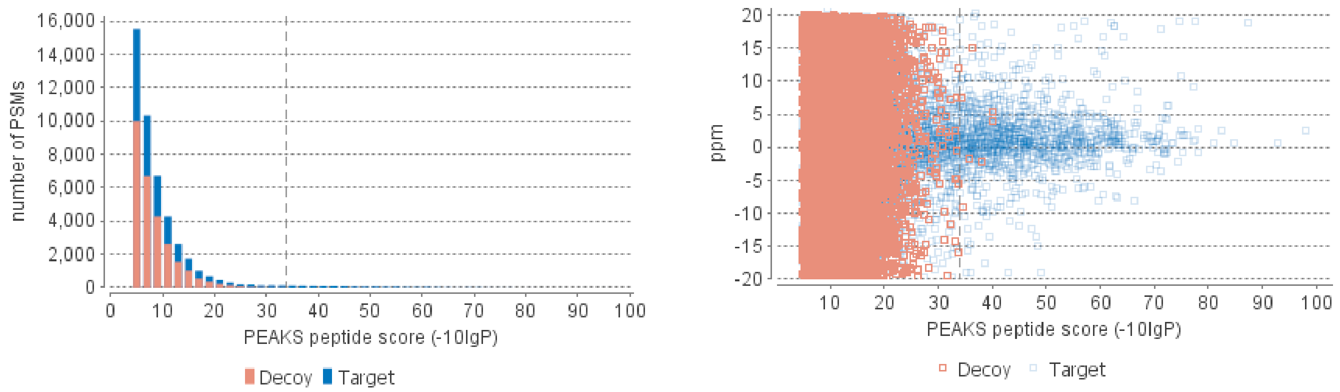

Table S8. Statistics of data.

	#Frames			#Features	#Precursors	Identified			#Peptides	#Sequences	#Proteins*		
	MS1	MS/MS	#Chimera			#PSMs	#Scans	#Features**			Groups	All	Top
Total	2381	23800	10356	44158	110274	1096	1094	546	744	681	199	587	479

0506_3_F1F2_UF_C18_Salmonella Curupira	2381	23800	10356	44158	110274	1096	1094	546	744	681	199	587	479

* proteins with significant peptides are used in counts.
** features are identified by DB search only.

Figure S9. Sample overlap for Proteins and Peptides (up to 8 samples). **(a)** All Proteins; **(b)** Top Proteins; **(c)** Peptides;

(a)	(b)	(c)
**Not applicable to only one sample**	**Not applicable to only one sample**	**Not applicable to only one sample**

Figure S10. Distribution of peptide feature detection. **(a)** Feature m/z distribution; **(b)** Feature RT distribution.

(a) **(b)**

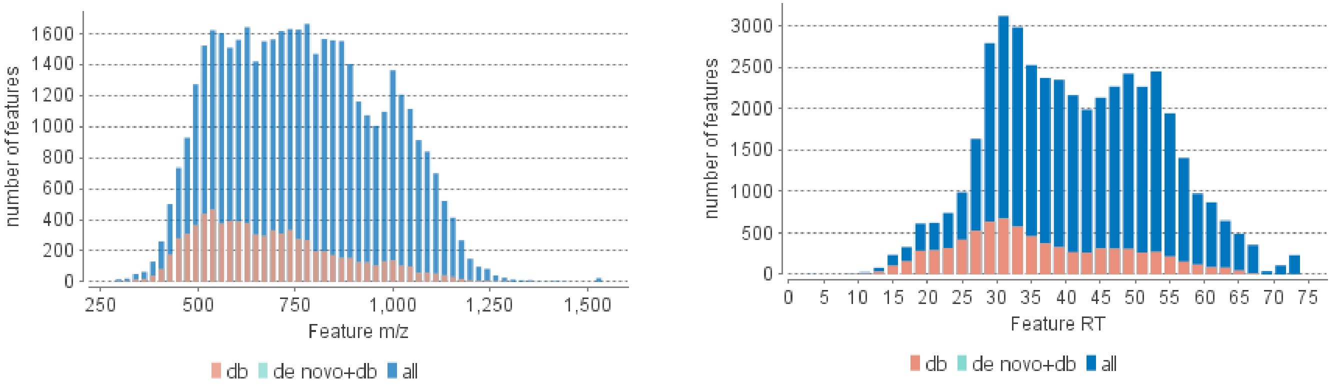

Figure S11. Distribution of identified peptide features. **(a)** Feature abundance distribution; **(b)** *De novo* sequencing validation.

(a) **(b)**

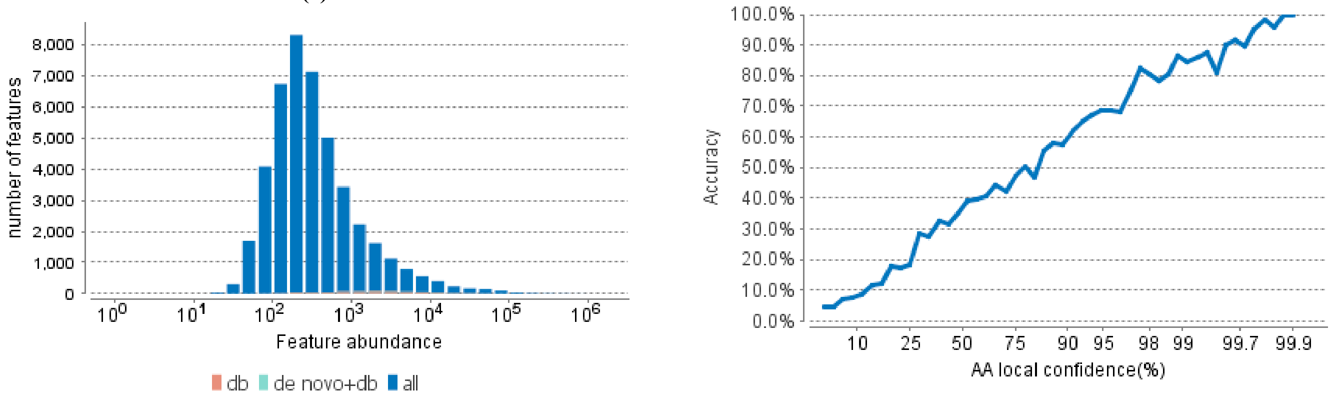

Table S9. Result filtration

parameters. Peptide -10lgP	≥33.9
PTM Ascore	≥95

Table S11. PTM profile.

Name	ΔMass	Position	#PSM	-10lgP	Abundance	AScore

Peptide mutation ion intensity	≥0%
Protein -10lgP	≥0
Proteins unique peptides	≥1
De novo score(%)	≥80%

Table S10. Statistics of filtered result.

FDR (Peptide-Spectrum Matches)	0.6%
FDR (Peptide Sequences)	0.9%
FDR (Protein Group)	3.5%
De Novo Only Spectra	159

Oxidation	15.99	M	80	71.76	6.89E3	1000.00
Deamidation	.98	NQ	35	63.53	6.8E3	1000.00
Acetylation	42.01	N-term	23	87.41	1.62E4	1000.00
Cysteinyl	119.00	C	9	58.12		1000.00
Citrullination	.98	R	4	56.73		35.44

3. Experiment Control

Figure S12. Precursor mass error of peptide-spectrum matches (PSM) in filtered result. **(a)** Distribution of precursor mass error in ppm; **(b)** Scatterplot of precursor m/z versus precursor mass error in ppm.

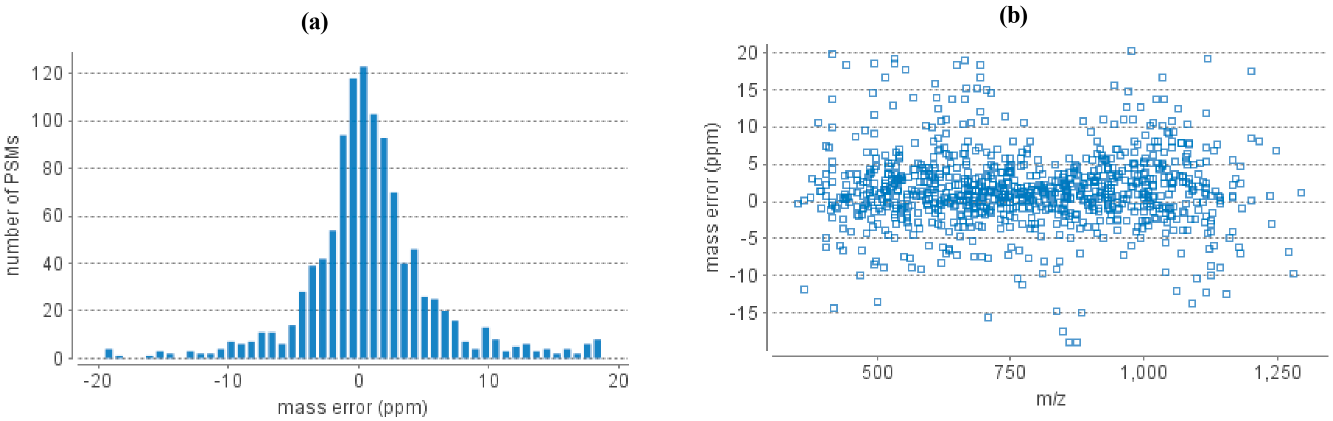

Table S12. Number of identified peptides in each sample by the number of missed cleavages.

Missed Cleavages	0	1	2	3	4+
0506_3_F1F2_UF_C18_Salmonella Curupira	0	0	0	0	744

4. Other Information

Table S13. Search parameters.

Query Type: Homology Match
Variable Modifications:
Oxidation (M): 15.99
Cysteinylation: 119.00

Table S14. Instrument parameters.

Fractions: 0506_3_F1-F2_UF_C18_Slot1-28_1_3732.d
Ion Source: ESI(nano-spray)
Fragmentation Mode: high energy CID (y and b ions)

Fragment ion tolerance: 0.1
L equals I: true
Q equals K: true
Report number: 1
Maximum # of PTMs: 2
De novo score(%) threshold: 15
Peptide hit threshold (-10logP): 30.0
Peaks run ID: 185
Merge Options: no merge
Precursor Options: corrected
Charge Options: no correction
Filter Charge: 1 - 6
Process: true
Associate chimera: yes

MS Scan Mode: TimsTOF
MS/MS Scan Mode: TimsTOF

1. Notes

2. Result Statistics

Figure S13. False discovery rate (FDR) curve. X axis is the number of peptides being kept. Y axis is the corresponding FDR.

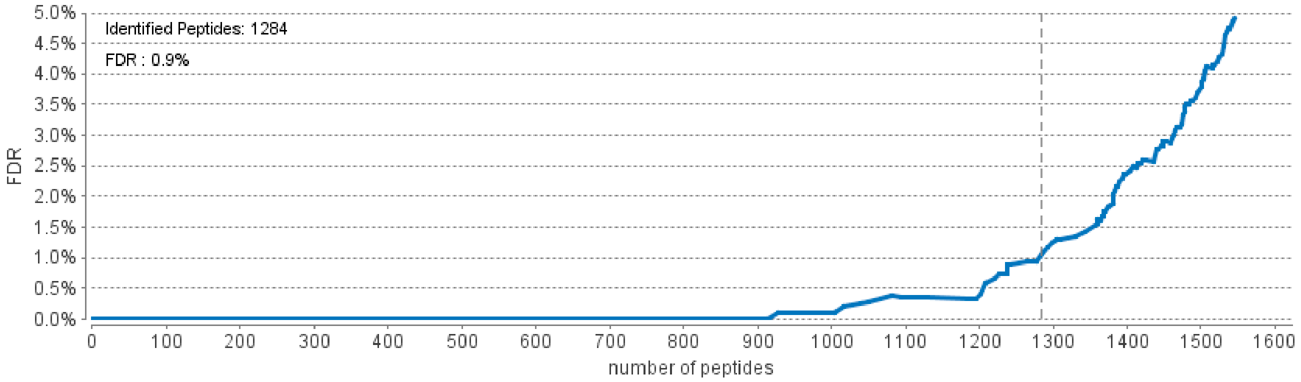

Figure S14. PSM score distribution. **(a)** Distribution of PEAKS peptide score; **(b)** Scatterplot of PEAKS peptide score versus precursor mass error.

(a) **(b)**

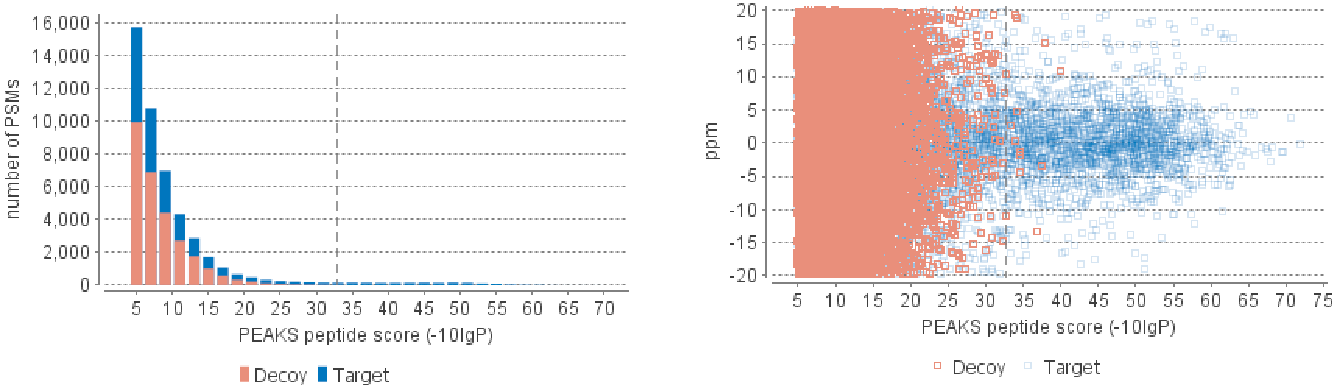

Table S15. Statistics of data.

	#Frames			#Features	#Precursors	Identified			#Peptides	#Sequences	#Proteins*		
	MS1	MS/MS	#Chimera			#PSMs	#Scans	#Features**			Groups	All	Top
Total	4966	22770	4138	25621	120006	1838	1837	745	1284	1220	248	756	621
0506_18_1_Salmonella_Manchas_C18	4966	22770	4138	25621	120006	1838	1837	745	1284	1220	248	756	621

* proteins with significant peptides are used in counts.
** features are identified by DB search only.

Figure S15. Sample overlap for Proteins and Peptides (up to 8 samples). **(a)** All Proteins; **(b)** Top Proteins; **(c)** Peptides;

(a)
Not applicable to only one sample

(b)
Not applicable to only one sample

(c)
Not applicable to only one sample

Figure S16. Distribution of peptide feature detection. **(a)** Feature m/z distribution; **(b)** Feature RT distribution.

(a)

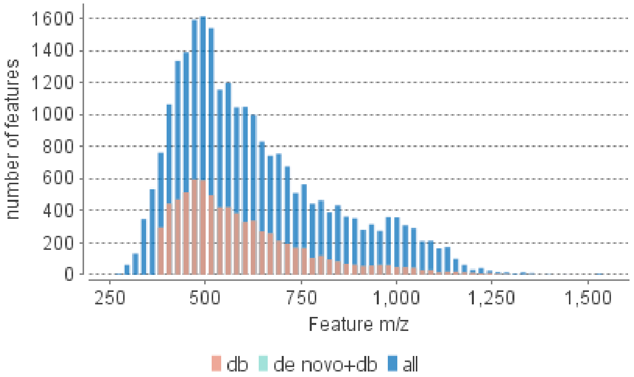

(b)

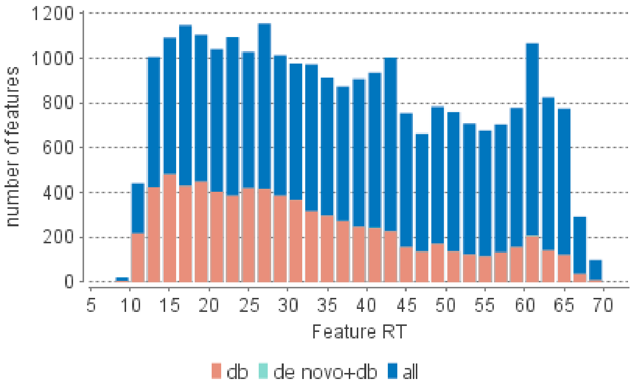

Figure S17. Distribution of identified peptide features. **(a)** Feature abundance distribution; **(b)** *De novo* sequencing validation.

(a)

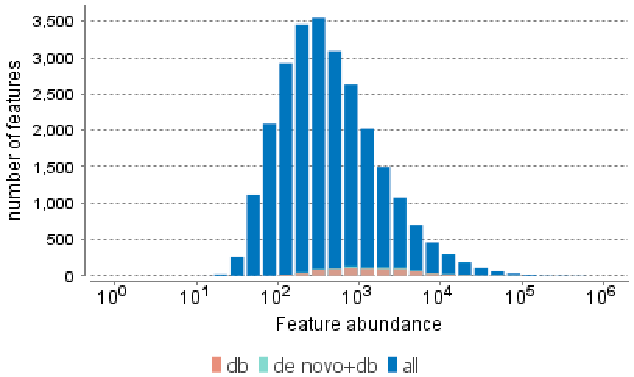

(b)

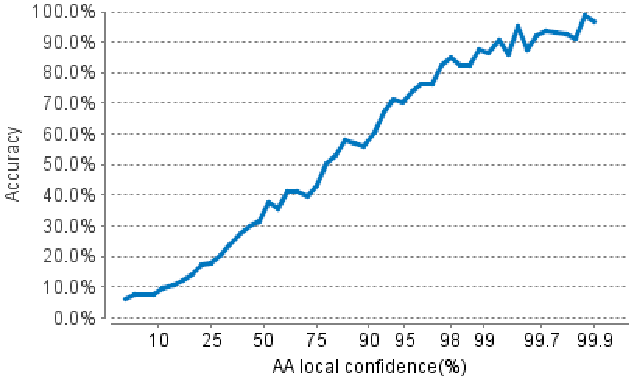

Table S16. Result filtration parameters.

Peptide -10lgP	≥32.8
PTM Ascore	≥95
Peptide mutation ion intensity	≥0%
Protein -10lgP	≥0

Table S18. PTM profile.

Name	ΔMass	Position	#PSM	-10lgP	Abundance	AScore
Oxidation	15.99	M	200	61.36	2.01E3	1000.00
Acetylation	42.01	N-term	67	62.53	2.19E3	1000.00

Proteins unique peptides	≥1
De novo score(%)	≥80%

Deamidation	.98	NQ	26	62.99		16.49
Cysteinyl	119.00	C	11	49.15	1.7E3	1000.00
Citrullination	.98	R	9	59.83	7.65E2	166.54
Ser->Glu	42.01	E	8	61.10	3.29E3	1000.00
Thr->Cys	1.96	C	4	42.92	3.81E4	1000.00
Tyr->Trp	23.02	W	4	42.92	3.81E4	1000.00

Table S17. Statistics of filtered result.

FDR (Peptide-Spectrum Matches)	0.7%
FDR (Peptide Sequences)	0.9%
FDR (Protein Group)	4.0%
De Novo Only Spectra	290

3. Experiment Control

Figure S18. Precursor mass error of peptide-spectrum matches (PSM) in filtered result. **(a)** Distribution of precursor mass error in ppm; **(b)** Scatterplot of precursor m/z versus precursor mass error in ppm.

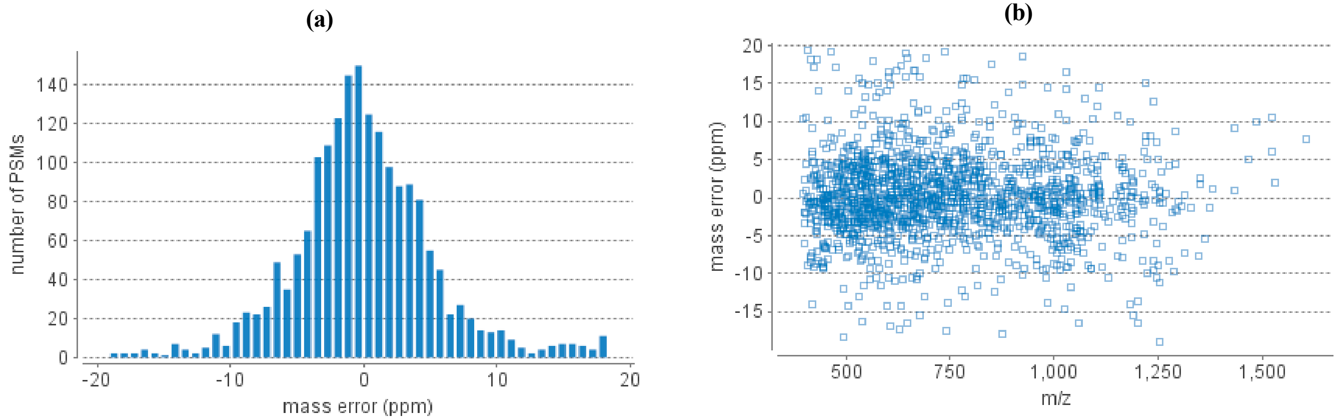

Table S19. Number of identified peptides in each sample by the number of missed cleavages.

Missed Cleavages	0	1	2	3	4+
0506_18_1_Salmonella_Manchas_C18	0	0	0	0	1284

4. Other Information

Table S20. Search parameters.

Query Type: Homology Match
Variable Modifications:
  Oxidation (M): 15.99
  Cysteinylation: 119.00
Fragment ion tolerance: 0.1
L equals I: true
Q equals K: true

Table S21. Instrument parameters.

Fractions: 0506_18_1_Salmonella C18_Slot1-1_1_10456.d
Ion Source: ESI(nano-spray)
Fragmentation Mode: CID, CAD(y and b ions)
MS Scan Mode: TimsTOF
MS/MS Scan Mode: TimsTOF

Report number: 1
Maximum # of PTMs: 2
De novo score(%) threshold: 15
Peptide hit threshold (-10logP): 30.0
Peaks run ID: 187
Merge Options: no merge
Precursor Options: corrected
Charge Options: no correction
Filter Charge: 1 - 6
Process: true
Associate chimera: yes
