# Supplementary material for: Immunopeptidomics of Salmonella enterica Serovar Typhimurium-Infected Pig Macrophages Genotyped for Class II Molecules
Source: Biology (Basel). 2024 Oct 16;13(10):832. doi: 10.3390/biology13100832 (PMC11505383; doi:10.3390/biology13100832)
Supplement: Supplementary file 1 [file biology-13-00832-s001.zip › Supplementary material S2.pdf]

Supplementary material S2

Table S22. Representation of the peptides obtained from a) pig 2 (Lr-0.12) and b) pig 3 (Lr-0.21), constructed from shared class II molecule sequences. The CORE (portion of a peptide in the alignment window) [46] is highlighted in bold in the peptide sequence, considering positions 1-9 identified in the elution motifs.

a) Pig 2 (Lr-0.12)

**CORE position**
**1 2 3 4 5 6 7 8 9**
RQTVAVGV**IKAVDKKAA**GAGKVTKSAQKAQK
AVRDMRQTVAVGV**IKAVDKKAA**GAGKVT
AVRDMRQTVAVGV**IKAVDKKAA**GAGKVTK
RQTVAVGV**IKAVDKKAA**GAGKVTKSAQKAQ
RQTVAVGV**IKAVDKKAA**GA
AVRDMRQTVAVGV**IKAVDKKAA**GAGKVTKSAQKA
AVRDMRQTVAVGV**IKAVDKKAA**GAGK
RQTVAVGV**IKAVDKKAA**GAGKVTKSAQKAQKAK
AVRDMRQTVAVGV**IKAVDKKAA**GAGKVTKSAQKAQK
RQTVAVGV**IKAVDKKAA**GAGKVTKS
AVRDMRQTVAVGV**IKAVDKKAA**GAGKV
AVRDMRQTVAVGV**IKAVDKKAA**GAGKVTKSA
RQTVAVGV**IKAVDKKAA**GAGKVT
AVRDMRQTVAVGV**IKAVDKKAA**GAGKVTKS
GVMVGMGQKDSY**VGDEAQSKR**
GMGQKDSY**VGDEAQSKR**
GVMVGMGQKDSY**VGDEAQSKR**GIL
GVMVGMGQKDSY**VGDEAQSKR**GIL
GMGQKDSY**VGDEAQSKR**GIL
TEHAKRKT**VTAMDVVYA**LKR
TEHAKRKT**VTAMDVVYA**LKRQGRTLYGFGG
TEHAKRKT**VTAMDVVYA**LKRQGRTLY
T**VTAMDVVYA**LKR
AKRKT**VTAMDVVYA**LKRQGRTLYGFGG

b) Pig 3 (Lr-0.21)

**CORE position**
**1 2 3 4 5 6 7 8 9**
V**ARPPKVQVY**SR
V**ARPPKVQVY**SRHPAENGK
V**ARPPKVQVY**
V**ARPPKVQVY**SRHPAENGKPN
V**ARPPKVQVY**SRHPAE

V**ARPPKVQVY**SRHPA
V**ARPPKVQVY**SRHPAEN
ISKQEY**DESGPSIVH**RKCF
EY**DESGPSIVH**R
ISKQEY**DESGPSIVH**RK
EY**DESGPSIVH**RK
ISKQEY**DESGPSIVH**R
DTKG**FFDPNTEEN**L
SDPSDDTKG**FFDPNTEEN**
**FFDPNTEEN**
SEILSDPSDDTKG**FFDPNTEEN**
SDPSDDTKG**FFDPNTEEN**LT
DDTKG**FFDPNTEEN**
ILSDPSDDTKG**FFDPNTEEN**L
SDPSDDTKG**FFDPNTEEN**L
ILSDPSDDTKG**FFDPNTEEN**
G**FFDPNTEEN**
SEILSDPSDDTKG**FFDPNTEEN**L
SEILSDPSDDTKG**FFDPNTEEN**LT

Table S23. Binding affinity prediction

**AFFINITY**					
ID	BINDERS <5%			UNIQUE BINDERS < 5%	
	MHC-CLAS II	SLA-DR	SLA-DQ	SLA-DR	SLA-DQ
PIG 1	979	345	634	25	71
PIG 2	10249	3145	7140	288	267
PIG 3	16526	11950	4576	469	640

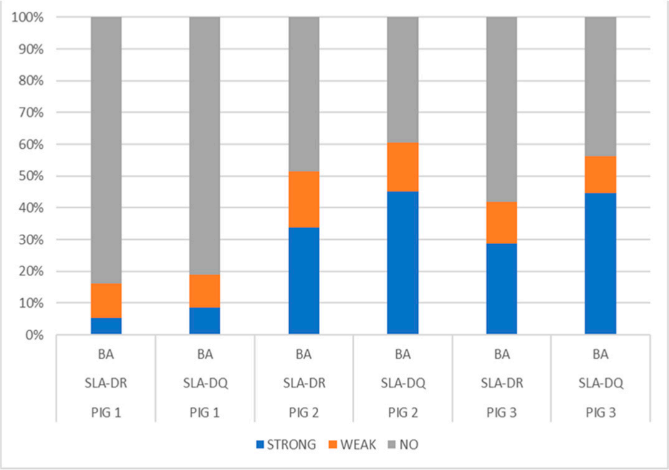

Figure S19. Binding affinity (BA) prediction distribution, considering total data percentages: strong BA (<1%rank), weak BA (<5%rank) and no binding BA (≥5%rank).

PIG	MHC-Class	SLA-DR	SLA-DQ
1 (Lr-0.23)	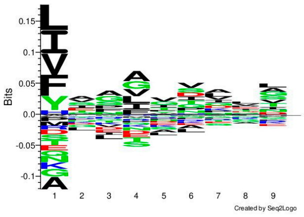	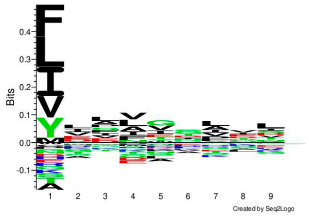	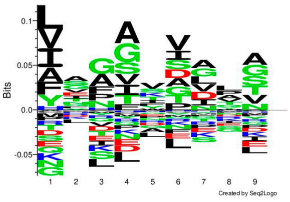
2 (Lr-0.21)	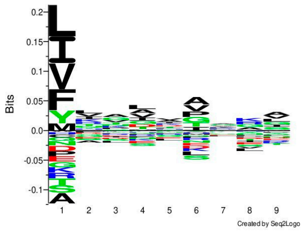	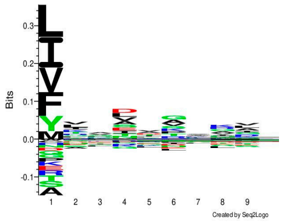	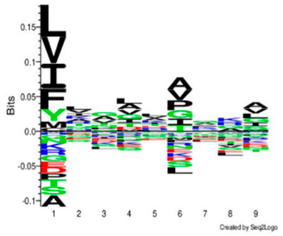

3

(Lr-0.12)

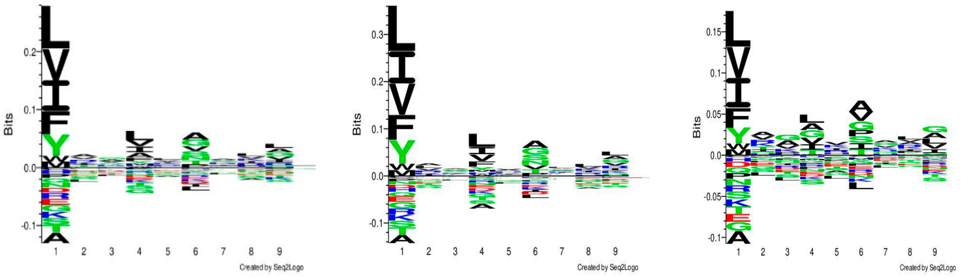

Figura S20. Logos performed with binding affinity data (<5% rank BA). Distribution considering a) MHC-class II, b) SLA-DR and c) SLA-DQ for each individual.
